# Supplementary material for: Evaluating pharmacological THRomboprophylaxis in Individuals undergoing superficial endoVEnous treatment across NHS and private clinics in the UK: a multi-centre, assessor-blind, randomised controlled trial—THRIVE trial
Source: BMJ Open. 2024 Feb 17;14(2):e083488. doi: 10.1136/bmjopen-2023-083488 (PMC10875503; doi:10.1136/bmjopen-2023-083488)
Supplement: Author's manuscript [file bmjopen-2023-083488.draft_revisions.pdf]

BMJ Open is committed to open peer review. As part of this commitment we make the peer review history of every article we publish publicly available.

When an article is published we post the peer reviewers' comments and the authors' responses online. We also post the versions of the paper that were used during peer review. These are the versions that the peer review comments apply to.

The versions of the paper that follow are the versions that were submitted during the peer review process. They are not the versions of record or the final published versions. They should not be cited or distributed as the published version of this manuscript.

BMJ Open is an open access journal and the full, final, typeset and author-corrected version of record of the manuscript is available on our site with no access controls, subscription charges or pay-per-view fees (http://bmjopen.bmj.com).

If you have any questions on BMJ Open's open peer review process please email info.bmjopen@bmj.com

BMJ Open

THRomboprophylaxis in Individuals undergoing superficial endoVEnous treatment: a multi-centre, assessor blind, randomised controlled trial – THRIVE trial

Journal:	*BMJ Open*
Manuscript ID	bmjopen-2023-083488
Article Type:	Protocol
Date Submitted by the Author:	20-Dec-2023
Complete List of Authors:	Machin, Matthew; Imperial College London, Section of Vascular Surgery, Department of Surgery and Cancer, Charing Cross Hospital; Imperial College Healthcare NHS Trust, Imperial Vascular Unit, St Mary's Hospital<br>Whittley, Sarah; Imperial College London, Section of Vascular Surgery, Department of Surgery and Cancer, Charing Cross Hospital<br>Norrie, John; The University of Edinburgh Usher Institute of Population Health Sciences and Informatics, Edinburgh Clinical Trials Unit, University of Edinburgh, Level 2, NINE Edinburgh BioQuarter<br>Burgess, Laura; Imperial College London, Section of Vascular Surgery, Department of Surgery and Cancer, Charing Cross Hospital<br>Hunt, Beverley; Guy's & St Thomas' Foundation Trust, Thrombosis & Haemophilia Centre, Westminster Bridge Road<br>Bolton, Layla; Imperial College Healthcare NHS Trust, Imperial Vascular Unit, St Mary's Hospital<br>Shalhoub, Joseph; Imperial College London, Section of Vascular Surgery, Department of Surgery and Cancer, Charing Cross Hospital; Imperial College Healthcare NHS Trust, Imperial Vascular Unit, St Mary's Hospital<br>Everington, Tamara; Hampshire Hospitals NHS Foundation Trust, Aldermaston Road, Basingstoke<br>Gohel, Manjit; Cambridge University Hospitals NHS Foundation Trust, Cambridge Vascular Unit, Addenbrookes Hospital, Hills Road<br>Whiteley, Mark S; University of Surrey<br>Rogers, Steven; The University of Manchester, Division of Cardiovascular Sciences, School of Medical Sciences, Faculty of Biology, Medicine and Health, Manchester Academic Health Science Centre; Manchester University NHS Foundation Trust, Manchester Academic Vascular Research and Innovation Centre (MAVRIC), Oxford Road<br>Onida, Sarah; Imperial College London, Academic Section of Vascular Surgery Department of Surgery & Cancer Imperial College London<br>Turner, Benedict; Imperial College London, Section of Vascular Surgery, Department of Surgery and Cancer, Charing Cross Hospital<br>Nandhra, Sandip; Newcastle University, Population Health Sciences Institute, Newcastle Upon Tyne<br>Lawton, Rebecca; Imperial College London, Academic Section of Vascular Surgery<br>Stephens-Boal, Annya; Imperial College London, Department of Surgery & Cancer<br>Singer, Carolyn; Charing Cross Hospital, Department of Surgery and Cancer

	Dunbar, Joanne; Charing Cross Hospital, Department of Surgery and Cancer<br>Davies, AH; Charing Cross Hospital, Department of Surgery and Cancer; St Mary’s Hospital<br>Carradice, Daniel; Hull York Medical School; Hull University Teaching Hospitals NHS Trust
Keywords:	VASCULAR SURGERY, Thromboembolism < CARDIOLOGY, Anticoagulation < HAEMATOLOGY

SCHOLARONE™
Manuscripts

Title: THRomboprophylaxis in Individuals undergoing superficial endoVEnous treatment: a multi-centre, assessor blind, randomised controlled trial – THRIVE trial

Authors: Matthew Machin[1,2], Sarah Whittley[1], John Norrie[3], Laura Burgess[1], Beverley J Hunt[4], Layla Bolton-Saghdaoui[2], Joseph Shalhoub[1,2], Tamara Everington[5], Manjit Gohel[6], Mark Whiteley[7], Steven Rogers[8,9], Sarah Onida[1,2], Benedict Turner[1], Sandip Nandhra[10], Rebecca Lawton[1], Annya Stephens-Boal[1], Carolyn Singer[1], Joanne Dunbar[1], Daniel Carradice[11], Alun Huw Davies[1,2].

[1] Section of Vascular Surgery, Department of Surgery and Cancer, Imperial College London, Charing Cross Hospital, London W6 8RF, United Kingdom.

[2] Imperial Vascular Unit, Imperial College Healthcare NHS Trust, St Mary's Hospital, London W2 1NY, United Kingdom.

[3] Usher Institute of Population Health Sciences and Informatics, Edinburgh Clinical Trials Unit, University of Edinburgh, Level 2, NINE Edinburgh BioQuarter, 9 Little France Road, Edinburgh, EH16 4UX, United Kingdom

[4] Thrombosis & Haemophilia Centre, Guy's and St Thomas' NHS Foundation Trust, Westminster Bridge Road, London, SE1 7EH, United Kingdom.

[5] Hampshire Hospitals NHS Foundation Trust, Aldermaston Road, Basingstoke, Hampshire, RG24 9NA, United Kingdom.

[6] Cambridge Vascular Unit, Addenbrookes Hospital, Cambridge University Hospitals NHS Foundation Trust, Hills Road, Cambridge, CB2 0QQ, United Kingdom.

[7] The Whiteley Clinic, 1 Chapel Place, London, W1G 0BG, United Kingdom

For peer review only - http://bmjopen.bmj.com/site/about/guidelines.xhtml

[8] Division of Cardiovascular Sciences, School of Medical Sciences, Faculty of Biology, Medicine and Health, University of Manchester, Manchester Academic Health Science Centre, Manchester, M13 9PL, United Kingdom.

[9]Manchester Academic Vascular Research and Innovation Centre (MAVRIC), Manchester University NHS Foundation Trust, Oxford Road, Manchester, M13 9WL, United Kingdom

[10] Population Health Sciences Institute, Newcastle University, Newcastle Upon Tyne, NEW1 7RU, United Kingdom.

[11] Academic Vascular Surgical Unit, Hull York Medical School, Allam Diabetes Centre, Hull Royal Infirmary, Anlaby Road, Hull, HU3 2JZ, United Kingdom

Professor Alun Davies and Mr Daniel Carradice should be considered joint senior author.

Corresponding author: Professor Alun H. Davies, Professor of Vascular Surgery, Section of Vascular Surgery, Department of Surgery and Cancer, Imperial College London, Charing Cross Hospital, London W6 8RF, United Kingdom. Email: a.h.davies@imperial.ac.uk.

For peer review only

ABSTRACT

Introduction

Endovenous therapy is the first-choice management for symptomatic varicose veins in NICE guidelines, with 56-70,000 procedures performed annually in the UK. Venous thromboembolism (VTE), including deep vein thrombosis (DVT) and pulmonary embolism (PE), and endothermal heat-induced thrombosis (EHIT), are known complications of endovenous therapy, occurring at a rate of up to 3.4%. In an attempt to reduce VTE, 73% of UK practitioners administer pharmacological thromboprophylaxis. However, no high-quality evidence to support this practice exists. Pharmacological thromboprophylaxis may have clinical and cost benefit in preventing VTE, however, further evidence is needed. The aims of this study are to establish whether when endovenous therapy is undertaken: a single dose or course of pharmacological thromboprophylaxis alters the risk of VTE; pharmacological thromboprophylaxis is associated with an increased rate of bleeding events; pharmacological prophylaxis is cost effective.

Methods and analysis

A multi-centre, assessor-blind, randomised controlled trial (RCT). We aim to recruit 6660 participants undergoing superficial endovenous interventions under local anaesthesia. Forty sites across the UK, both NHS and private, will be included. Participants will be randomised to either intervention (a single dose or extended course of pharmacological thromboprophylaxis plus compression) or control (compression alone). Participants will undergo a lower limb venous duplex ultrasound scan at 21-28 days post-procedure to identify asymptomatic DVT. The ultrasound duplex scan will be conducted locally by blinded assessors. Participants will also be contacted remotely for follow-up at 7-days and 90-days post-procedure. The primary outcome is imaging confirmed lower limb DVT with or without symptoms, or PE with symptoms within 90 days of treatment. The main analysis will be according to

For peer review only - http://bmjopen.bmj.com/site/about/guidelines.xhtml

the intention-to-treat principle and will compare the rates of VTE at 90 days, using a repeated measures analysis of variance (ANOVA), adjusting for any pre-specified strongly prognostic baseline covariates using a mixed effects logistic regression.

Trial registration number

ISRCTN18501431

For peer review only

KEYWORDS

Venous thromboembolism, deep vein thrombosis, pulmonary embolism, superficial endovenous treatment, thromboprophylaxis, low molecular weight heparin, direct-acting oral anticoagulants.

ARTICLE SUMMARY

Strengths and limitations of this study

- The study will serve as a large, randomised controlled trial providing grade A evidence on the most clinically- and cost-effective thromboprophylaxis regimen following superficial endovenous treatment.
- The primary outcome holds clinical significance.
- Using VTE prophylaxis may be associated with adverse clinical outcomes, increased risks and may not be cost-effective.

For peer review only - http://bmjopen.bmj.com/site/about/guidelines.xhtml

- Should pharmacological thromboprophylaxis be shown to offer no additional benefit to patients undergoing superficial endovenous intervention, stopping this practice has the potential to generate significant cost savings for healthcare providers.

Word count: 3,992

For peer review only

INTRODUCTION

Varicose veins, also known as superficial refluxing veins, affect up to 45% of the UK population (1). Varicose veins not only reduce physical and mental health-related quality of life, but also contribute significantly to chronic venous disease, which is responsible for over half of all cases of leg ulcers (2–

For peer review only - http://bmjopen.bmj.com/site/about/guidelines.xhtml

4). Superficial endovenous treatment (SET), offers a minimally invasive approach that can conveniently be performed in an outpatient setting (5). Endovenous surgery stands as the recommended first-choice management for symptomatic varicose veins, in line with NICE guidelines [CG168] (5). This recommendation aligns with the European Society for Vascular Surgery Clinical Practice Guidelines, providing a level I recommendation for endovenous techniques (6). SET not only improves quality of life and facilitates venous ulcer healing, but also offers cost savings to healthcare providers (7–13). Annually, approximately 30,000 endovenous varicose vein procedures are carried out within the NHS, a number estimated to reach 68,800 with full adherence to NICE guidelines (14). Additionally, an estimated 30,000 to 40,000 procedures are undertaken annually in the private sector.

Despite its efficacy, SET is associated with thrombotic complications, presenting occurrences of venous thromboembolism (VTE) and endothermal heat-induced thrombosis (EHIT) at rates as high as 3.4%. VTE, encompassing deep vein thrombosis (DVT) and pulmonary embolism (PE), is a significant cause of disability and subsequent societal economic consequences (15). Hospital-acquired thrombosis (HAT), which is defined as the development of VTE within 90 days of a hospital episode, significantly contributes to morbidity and mortality, with statistics indicating a rate of 57 deaths per 100,000 admissions within the NHS (16). Complications following a DVT are substantial, with up to 50% of patients developing post thrombotic syndrome (PTS), characterised by chronic leg pain, oedema and skin changes (17,18). Additionally, PE is associated with lifelong functional and psychological repercussions, ultimately posing a risk of death during the index event (19,20). EHIT, encompassing any thrombus forming within 4 weeks of endovenous ablation and extending from the treated vein toward or into a deep vein, is categorised into four classes. EHIT classes 3 – 4 involve significant thrombus extension into and encompassing the deep vein, often necessitating treatment similar to that for DVT (9,10). SET presents a unique VTE risk compared to other short-stay surgical procedures, exhibiting a VTE rate comparable to that observed in major joint surgeries (16).

For peer review only - http://bmjopen.bmj.com/site/about/guidelines.xhtml

Comparable day-case surgical procedures such as inguinal hernia repair or laparoscopic cholecystectomy exhibit a notably lower VTE rate of 0.3% (21).

In attempting to reduce the risk of VTE, UK clinicians exhibit varying approaches: 52% routinely prescribe a single dose of low-molecular weight heparin (LMWH) and 15% routinely prescribe extended pharmacological thromboprophylaxis with either LMWH or a direct-acting oral anticoagulant (DOAC), while 33% do not prescribe any pharmacological thromboprophylaxis (22). These practices align with findings from a 2019 national survey of vascular surgeons in Ireland, indicating that 73% of practitioners routinely prescribe pharmacological thromboprophylaxis for SET, utilising either a single dose of LMWH or extended prophylaxis, while 27% of practitioners do not prescribe any form of pharmacological thromboprophylaxis (23). Contradicting NICE guidelines, the routine use of pharmacological thromboprophylaxis for SET has become prevailing practice, despite having no supportive evidence base. A systematic review and meta-analysis published in 2022 failed to identify any high-quality evidence to support current pharmacological thromboprophylaxis strategies in this group of patients (24). However, the study suggested there is a significant reduction in the rate of DVT with additional pharmacological thromboprophylaxis (1.09% versus 3.20% for pharmacological thromboprophylaxis versus compression alone). International and national guidelines reflect the paucity of evidence in this area, with the European Society of Vascular Surgery guidelines providing a (IIa B) recommendation for consideration of individualised thromboprophylaxis strategies (6). This is exacerbated by NICE NG89 recommending that “prophylaxis is generally not needed for people undergoing varicose vein surgery” if their VTE risk assessment deems them low-risk (25).

Clinicians also lack confidence in current risk assessment tools (RATs) for patients undergoing SET (15). Despite the utilisation of RATs such as the Department of Health Risk Assessment (DHRA) Tool in the UK and the Caprini RAT in Europe and the US, none have undergone validation for varicose vein procedures. Consequently, varicose vein intervention-specific RATs have emerged, yet these,

For peer review only

too, lack validation. Thus, there is currently no consensus on which risk factors provide clinical indication for pharmacological thromboprophylaxis or confer high-risk for VTE (26).

Pharmacological thromboprophylaxis presents potential clinical and cost benefits in VTE prevention, however grade A evidence is required to either support or refute this practice. Furthermore, as current RATs have not been validated in this patient group, unbiased prospective evidence will help guide risk-stratifying patients in the future. This research question also aligns with the James Lind Alliance priority setting for venous disease, further underlining the need for this trial.

Objectives

The aims of this study are to establish whether in patients undergoing SET:

1) A single dose of pharmacological thromboprophylaxis decreases the risk of VTE
2) An extended course of pharmacological thromboprophylaxis decreases the risk of VTE
3) Pharmacological thromboprophylaxis is associated with an increased rate of bleeding events
4) Providing pharmacological prophylaxis is cost-effective
5) Any pharmacological thromboprophylaxis affects the rate of VTE

METHODS AND ANALYSIS

Trial design

For peer review only - http://bmjopen.bmj.com/site/about/guidelines.xhtml

This is a multi-centre, assessor-blind randomised controlled trial with a superiority comparison. The primary outcome is assessed blindly.

Study setting

This trial will take place in NHS hospitals and private clinics delivering endovenous varicose vein procedures under local anaesthesia. Recruitment centres will need to have a pre-existing practice prior to the trial to prevent any learning curve effects.

Eligibility criteria

Inclusion criteria are: adult patients (>18 years) scheduled to undergo endovenous intervention of truncal varicose veins under local anaesthesia. Treatment technologies include radiofrequency, laser, mechanochemical, foam sclerotherapy and cyanoacrylate glue.

Exclusion criteria are: clinical indication for therapeutic anticoagulation (e.g. atrial fibrillation, previous personal or first-degree relative history of VTE, thrombophilia). Also female patients of childbearing potential with a positive pregnancy test; those with a history of allergy to heparins or DOACs; a history of heparin-induced thrombocytopenia in the last year; inherited and acquired bleeding disorders; evidence of active bleeding; concomitant major health problems such as active cancer and chronic renal and/or liver impairment; known thrombocytopenia (platelets known to be less than 50 x 10^9/l); surgery or major trauma in the previous 90 days; recent ischaemic stroke in the previous 90 days; inability to provide consent.

Interventions

For peer review only - http://bmjopen.bmj.com/site/about/guidelines.xhtml

There are currently three thromboprophylaxis strategies used across the UK, with the trial arms of this application mirroring these practices (23). Intervention arms will consist of a single prophylactic dose of LMWH (e.g. dalteparin sodium, tinzaparin sodium, enoxaparin sodium) plus compression as per local practice (e.g. stockings, bandages, wraps, pads), and a single dose of LMWH plus extended thromboprophylaxis with LMWH or a DOAC (e.g. rivaroxaban, apixaban, dabigatran etexilate) plus compression. The choice between LMWH or DOAC for the extended thromboprophylaxis arm will be site-specific and dependent on local practice. The duration of this must be at least 7 days, but can be in line with local practice i.e., between 7 and 14 days in duration. The control arm will consist of compression (bandages, stockings, wraps or padding) as per local practice alone. The participant flow in the trial is displayed in *Figure 1*.

For peer review only

Primary outcome

Imaging confirmed lower limb DVT with or without symptoms, or PE with symptoms within 90 days of varicose vein treatment.

Secondary outcomes

The secondary outcomes include:

1) Individual components of the composite outcome
2) Comparisons of quality of life at 7- and 90-days post-procedure using the EQ-5D
3) Mortality rates in each group
4) Cost-effectiveness of providing pharmacological thromboprophylaxis
5) Exploratory analyses to assess how well the DHRA tool and Caprini score predict outcome

For peer review only - http://bmjopen.bmj.com/site/about/guidelines.xhtml

Safety outcomes

Safety monitoring includes any bleeding event. Major bleeding is defined as per the International Society on Thrombosis and Haemostasis standardised definition (27), which includes:

1) Bleeding into a critical organ
2) Bleeding into a surgical site requiring re-operation
3) Bleeding that leads to presentation to acute service

Sample size and study duration

The most comprehensive evidence from 52 studies suggests that the rate of VTE (encompassing DVT, PE, EHIT) post-endovenous great saphenous vein interventions is 1.7% (1). However, this analysis did not investigate treatment effects. To address this, we conducted an analysis of 229 study arms, pooling data from 480,581 participants undergoing endovenous interventions. Our approach involved subgroup analyses, comparing those receiving pharmacological thromboprophylaxis and compression versus compression alone, and distinguishing between asymptomatic screen-detected and symptomatic VTE (24).

For individuals receiving pharmacological thromboprophylaxis (either single dose of LMWH or extended duration), rates of DVT, PE, and EHIT 3 - 4 were 0.521%, 0.216%, and 0.354%, respectively, resulting in a maximum summary VTE rate of 1.09%. Those receiving compression alone showed rates of 2.264% for DVT, 0.058% for PE, and 0.878% for EHIT 3- 4, resulting in a maximum summary VTE rate of 3.20%. Rates of EHIT and DVT are distinct in this calculation, thus the impact of double counting contributing events will be negligible. In the subset data from RCT arms alone, rates of DVT were similar. When interpreting these figures in the context of the wider literature, balancing this with confounding by indication, and acknowledging that VTE rate will likely be lower in the compression alone arm due to the exclusion of individuals at the highest risk of VTE, we anticipate that the true

For peer review only - http://bmjopen.bmj.com/site/about/guidelines.xhtml

value lies nearer to 1.0% in the pharmacological thromboprophylaxis arm and 2.7% in the compression alone arm.

At 90% power and 2.5% alpha (to approximately control overall alpha to 5%, with two active drugs being compared with a common control), the study could detect a significant change of 1.7% in 90-day VTE. This base case would require 1554 participants per group. Allowing for 10% crossover (which can only be control patients, compression only, receiving pharmacological thromboprophylaxis, since everyone receives compression) increases this to 1919 per group. Allowing for a single interim analysis at half time (50% randomised with 90 day follow up) for early stopping, for either futility or overwhelming evidence of efficacy analysis, inflates this to 1998 per group, under a two-sided, asymmetric, group sequential design implementing the non-binding Hwang-Shih-DeCani spending function [lower bound (futility) with gamma-2; upper bound (efficacy) with gamma= -4] (28). If we then allow 10% for loss to follow up, the total sample, randomised 1:1:1 between all groups, becomes 6660 (or 2220 per group).

We will more accurately estimate the required sample size by simulation in a sample size re-estimation step at around 20% mature data, using Dunnett's 3-arm design with a common control, with correction, but for simplicity retaining the 1:1:1 equal randomisation; and inputting the observed missing data proportion at that stage.

Interim analysis

There will be a formal interim analysis with the possibility of stopping early for futility (no prospect of a clinically meaningful treatment effect), at the point of 50% mature primary outcome data. Full details of the stopping boundaries and analysis will be detailed in a Statistical Analysis Plan (SAP), which the independent Data Monitoring Committee (iDMC) will approve prior to seeing unblinded data. The unblinded statistician will have no other role in the study while it is ongoing. The stopping

For peer review only - http://bmjopen.bmj.com/site/about/guidelines.xhtml

rules are statistically non-binding. The iDMC may recommend early stopping of the study if the boundaries are crossed. They would make a recommendation to the independent Trial Steering Committee (TSC) who may or may not endorse that recommendation. The trial may stop at any time for safety if there is an excess of events in the intervention groups that is considered to generate avoidable harm. This decision would not be based on any statistical criterion and would be taken by the iDMC, then endorsed by the TSC.

Recruitment and randomisation

Adults scheduled to undergo SET will be pre-screened by a member of the direct care team and invited to discuss the trial with a member of the research team. Informed consent will be obtained from each participant prior to participation in the trial, following adequate explanation of the aims, methods, anticipated benefits, and potential hazards of the trial. The individual obtaining consent will be a registered healthcare professional and will have been delegated this duty by the Principal Investigator (PI) on the delegation log. Participants may provide their consent to participate in the trial electronically or in written form. Participants will initial each statement on the consent form to confirm agreement. A copy of the consent form will be provided to the participant for their records. There is no defined timeframe between initial consent and the baseline visit, however confirmation that the participant's consent is still valid prior to randomisation will be required in the baseline case report form (CRF).

Participants (n = 6,660) will undergo 1:1:1 web-based randomisation to one of three thromboprophylaxis strategies prior to undergoing SET. Randomisation will be conducted by an automated system linked to the Research Electronic Data Capture (REDCap) database setup via the Study Data Centre at the Edinburgh Clinical Trials Unit (ECTU), University of Edinburgh (a fully registered UKCRC Clinical Trials Unit, registration number 15). Recruitment will commence on 01 January 2024 for 27 months. The study will close on 31 December 2026.

For peer review only - http://bmjopen.bmj.com/site/about/guidelines.xhtml

For peer review only

Blinding

Clinicians and participants will be aware of their treatment allocation. Assessors, being those who perform the venous duplex ultrasound scan and those responsible for collecting follow-up data at 7- and 90- days post-procedure, will be blinded to the treatment allocation.

Follow-up periods

Participants will undergo a lower limb venous duplex ultrasound scan at 21 – 28 days post-intervention to identify asymptomatic DVT. This is timed to capture the peak onset of events which is at 3 weeks (29). Participants will be further followed up remotely by telephone, online or short message service (SMS) at 7- and 90-days with an expected VTE capture >95% (29). Longer term follow-up may be considered with award from a subsequent project grant to assess long-term efficacy of the intervention, hence contact at a later point will be included in the consenting process.

Data collection and confidentiality

Participant data will be entered into the REDCap database by the local research teams. Source data stored at study centres will be archived locally as per local study operating procedures. Data and all appropriate documentation will be stored for a minimum of 10 years after the completion of the study, including the follow-up period. Details of procedures for CRF completion will be provided in a separate study manual. A formal Data Management Plan will be constructed to describe the procedures involved in the data management activities and processes for the study so that it is managed and maintained in accordance with the Good Clinical Practice (GCP) guidelines, local Research and Governance Integrity Team (RGIT) standard operating procedures, appropriate regulatory requirements, and the study protocol.

For peer review only - http://bmjopen.bmj.com/site/about/guidelines.xhtml

For peer review only

Statistical analyses

All statistical analyses will be governed by a comprehensive SAP, written by the study statistician, and agreed by the TSC and iDMC before any unblinded data is seen. The main analysis will be according to the intention-to-treat principle where all consented participants will be included in the analysis retained in the group to which they were allocated (i.e., "as randomised") and for whom outcome data are available. All results will be presented as point estimates, confidence intervals at the appropriate level and associated p-values. Absolute measures of effect will be presented alongside relative measures. The primary analysis will compare the incidence of VTE at 90 days, using a repeated measures analysis of variance (ANOVA), adjusting for any pre-specified strongly prognostic baseline covariates using a mixed effects logistic regression. Study site will be included in the model as a random effect and pre-specified baseline covariates strongly related to outcome will be included to adjust the estimated treatment effect. The findings will be assessed for robustness against any missing data, first using multiple imputation assuming this data is missing at random and, if appropriate and the data permits, further sensitivity analyses will be attempted under any plausible missing data mechanisms not missing at random. Secondary outcomes will be analysed in a similar fashion with generalised linear models appropriate to the distribution of the outcome. Safety data will be summarised descriptively.

Internal pilot

There will be an internal pilot to assess feasibility of recruitment over nine months of recruitment to the trial, in which we will start recruiting from the (minimum) 40 centres. Site setup will be

For peer review only - http://bmjopen.bmj.com/site/about/guidelines.xhtml

staggered over nine months, i.e., five centres per month. The target number of participants by the end of the 9-month internal pilot is 1450 participants. For the internal pilot, we will use stop-go criteria based on a Green-Amber-Red statistical approach (see *Table 1*).

Progression criteria	Red	Amber	Green
% Threshold	<10.9%	10.9 – 20.9%	≥21%
Trial recruitment (of eligible participants)	<15%	15 – 17.6%	≥17.6%
Recruitment rate / site / month	<5	5 – 7.1	≥7.2
Number of centres opened	<25	25 – 40	≥40
Total number of participants recruited	<725	725 – 1449	≥1450

Table 1. Internal pilot of feasibility assessment at 9 months.

For peer review only

Cost-effectiveness analysis

Two health economic analyses will be conducted, and a separate Health Economics Analysis Plan (HEAP) will be developed by the Health Economist detailing the proposed analyses. The main analyses will be performed from the perspective of the NHS and Personal Social Services, with secondary analyses from a societal perspective.

A within-trial analysis will compare the two pharmacological thromboprophylaxis strategies to compression alone over the 90 days of the study. Resource use items associated with treatments in primary and secondary care will be collected using case notes and self-completed patient diaries, and costed using manufacturer list prices, previous literature, and national reference costs. Days off work and normal activities and other patient-related costs will be collected for a secondary analysis. EQ-5D will be collected at baseline and follow up, analysed using the NICE approved tariff.

If the trial indicates that pharmacological thromboprophylaxis could be an effective therapy, a Markov (state–transition) decision model will be constructed to compare the cost effectiveness of the two pharmacological thromboprophylaxis strategies and compression alone over a longer time horizon. The time horizon of the model will be 2-years, allowing extrapolation of sequelae of VTE

For peer review only - http://bmjopen.bmj.com/site/about/guidelines.xhtml

events (such as PTS) over the longer term to quantify the impact of VTE on patient health via quality adjusted life years (QALYs) and resource use. A preliminary model has been constructed based on published literature to identify the key variables that would need to be collected during the clinical study, and to estimate the number needed to treat (NNT) to avoid one VTE, above which pharmacological thromboprophylaxis would not be considered cost-effective at NICE thresholds. This model conservatively assumes 30% of patients with VTE develop PTS, with 3% of those having severe PTS.

The minimum cost of purchasing 10-days of thromboprophylaxis and providing allocated time for administration training equates to ~£63.13 (30,31). Our model assumes cost of treatment of VTE, non-severe and severe PTS, as £451, £872 and £1,547 respectively and estimates of the respective utility decrement associated with symptomatic VTE and PTS are 0.8628, 0.7745 and 0.6752 respectively (32). Different durations of thromboprophylaxis will be modelled. These estimates will be reviewed at the time of the cost-effectiveness analysis, and any changes to these estimates will be updated.

Utilising a 2-year time horizon, incremental cost effectiveness ratio of pharmacological thromboprophylaxis in comparison to no pharmacological therapy would be £13,339 per QALY if the NNT were 59 participants (1/0.017). For pharmacological thromboprophylaxis to be cost effective at a NICE willingness to pay threshold of £20,000 per QALY, the NNT would need to be below 80.

Main analyses will be undertaken from perspective of the NHS and Personal Social Services, with secondary analyses from a societal perspective. Health economic analysis will be conducted according to NICE reference case and CHEERS guidelines, including sensitivity analyses and probabilistic sensitivity analyses (33,34). Results will be presented as estimates of mean incremental costs, effects, and incremental cost per QALY.

For peer review only - http://bmjopen.bmj.com/site/about/guidelines.xhtml

Data monitoring, safety and quality control

An independent TSC and iDMC have been convened. The role of the TSC is to provide overall supervision of trial conduct and progress, and ensure the study adheres to GCP principles. The role of the iDMC is to oversee the safety of the trial participants, and the iDMC will be the only oversight committee that sees unblinded data as the trial progresses, which they will keep in strict confidence. Details of membership, responsibilities and frequency of meetings for the TSC and iDMC have been defined in separate Charters.

The Trial Management Group will oversee trial progress. All adverse events (AEs) will be collected and recorded on the REDCap database. Causality and relatedness will be assigned by the local PI or other appropriately trained and delegated individual. Research sites will inform the Coordinating Centre of all serious adverse events (SAEs) within 24 hours of knowledge of the event.

A study-specific risk assessment has been prepared in preparation for the study by the Trial Manager and study sponsor, which will be updated as required during the course of the trial. The frequency, type and intensity of monitoring visits will be detailed in a separate Data Monitoring Plan (DMP). The DMP will also detail the procedures for completion and sign-off of monitoring reports. In the event of a request for a trial site inspection by any regulatory authority, the study coordinating team must be notified as soon as possible. Participating investigators must agree to allow trial related monitoring, including audits, Research Ethics Committee (REC) review and regulatory inspections, by providing access to source data and other trial related documentation as required. Participant consent for this must be obtained as part of the informed consent process for the trial. The trial coordinating centre will centrally review eCRF data for errors and missing key data points on an ongoing basis.

Quality Control will be performed according to Imperial College internal procedures. The study may be audited by a Quality Assurance representative of the Sponsor. All necessary data and documents will be made available for inspection. The study may be subject to inspection and audit by regulatory

For peer review only - http://bmjopen.bmj.com/site/about/guidelines.xhtml

bodies to ensure adherence to GCP and the UK Policy Framework for Health and Social Care Research.

Patient and public involvement

Regular sustained patient and public involvement (PPI) has been undertaken to design and guide the study. Successful focus groups and interviews were held with patient representatives longitudinally throughout the trial development to gain insights into patients' lived experiences and gather feedback on potential trial designs. Online surveys were also conducted with the wider venous community to inform the Plain English Summary. The patient-facing documentation has been designed in collaboration with three patient representatives. Two patient advisers have agreed to sit on the TSC, as well as review updated patient-facing documentation and assist with the dissemination of study results. An online survey of key stakeholders was also undertaken at the UK's largest national conference to ascertain acceptability among clinicians of the proposed trial design.

For peer review only

ETHICS AND DISSEMINATION

The Study Coordination Centre has obtained approval from the London Brent REC (23/LO/0261). Protocol amendments will be submitted to the sponsor for review before applying for approval from the REC, Health Research Authority (HRA) and Medicines and Healthcare Products Regulatory Agency (MHRA) and updating the ICRCTN record accordingly. The study must also receive confirmation of capacity and capability from each participating NHS Trust before accepting any research activity is carried out. The study will be conducted in accordance with the

For peer review only - http://bmjopen.bmj.com/site/about/guidelines.xhtml

recommendations for physicians involved in research on human subjects adopted by the 18th World Medical Assembly, Helsinki 1964 and later revisions. Study findings will guide international clinical practice and stimulate key updates to international guidelines. Results will be published in high-impact journals alongside presentation at national and international vascular and haematology societies.

Funding statement

This work is supported by the National Institute for Health and Care Research (NIHR) Health Technology Assessment (HTA) programme (NIHR152877). The views expressed are those of the author(s) and not necessarily those of the NIHR or the Department of Health and Social Care.

Current study status

The study is currently in the process of setting up recruiting centres.

Trial sponsor

Imperial College London is the main sponsor for this study. Delegated responsibilities are assigned to the NHS Trusts/Health Boards and non-NHS sites taking part in this study.

Availability of data and materials

Data will be made available on reasonable request.

Competing interests

The authors have no competing interests to declare.

For peer review only - http://bmjopen.bmj.com/site/about/guidelines.xhtml

Author contributions

The author contributions were as follows: Conception (AHD, DC and MM); Design of the work (AHD, DC, MM, SW, JN, LB, BJH, LB-S, JS, TE, MG, MW, SR, SO, BT, SN, RL, AS-B, CS and JD); Drafting the first version of the manuscript (SW); Critical review of the manuscript (AHD, DC, MM, JN, LB, BJH, LB-S, JS, TE, MG, MW, SR, SO, BT, SN, RL, AS-B, CS and JD).

For peer review only

Abbreviations

AE	Adverse event
ANOVA	Analysis of variance
CRF	Case report form
DHRA	Department of Health Risk Assessment
DOAC	Direct-acting oral anticoagulant
DVT	Deep vein thrombosis
ECTU	Edinburgh Clinical Trials Unit
EHIT	Endothermal heat-induced thrombosis
GCP	Good clinical practice
HAT	Hospital-acquired thrombosis
HEAP	Health economics analysis plan
HRA	Health Regulatory Authority
HTA	Health Technology Assessment
ICER	Incremental cost-effectiveness ratio
iDMC	Independent Data Monitoring Committee

For peer review only - http://bmjopen.bmj.com/site/about/guidelines.xhtml

LMWH	Low-molecular weight heparin
MHRA	Medicines and Healthcare Regulatory Agency
NHS	National Health Service
NICE	National Institute for Care and Excellence
NIHR	National Institute for Health Research
NNT	Number needed to treat
PE	Pulmonary embolism
PI	Principal Investigator
PPI	Patient and public involvement
PTS	Post-thrombotic syndrome
QALY	Quality adjusted life years
RAT	Risk assessment tool
RCT	Randomised controlled trial
REDCap	Research Electronic Data Capture
REC	Research Ethics Committee
SAE	Serious adverse event
SAP	Statistical analysis plan
SMS	Short message service
TSC	Trial Steering Committee
UK	United Kingdom
VTE	Venous thromboembolism

For peer review only

Figure legends

For peer review only - http://bmjopen.bmj.com/site/about/guidelines.xhtml

Figure 1. THRIVE study flow chart

Abbreviations: DHRA, Department of Health Risk Assessment; DOAC, direct-acting oral anticoagulant; DVT, deep vein thrombosis; ICER, incremental cost-effectiveness ratio; LMWH, low-molecular weight heparin; VTE, venous thromboembolism.

REFERENCES

1. Salim S, Machin M, Patterson BO, Onida S, Davies AH. Global Epidemiology of Chronic Venous Disease: A Systematic Review With Pooled Prevalence Analysis. Annals of Surgery. 2021 Dec;274(6):971.

2. Carradice D, Mazari F a. K, Samuel N, Allgar V, Hatfield J, Chetter IC. Modelling the effect of venous disease on quality of life. Br J Surg. 2011 Aug;98(8):1089–98.

3. Kurz X, Lamping DL, Kahn SR, Baccaglini U, Zuccarelli F, Spreafico G, et al. Do varicose veins affect quality of life? Results of an international population-based study. J Vasc Surg. 2001 Oct;34(4):641–8.

4. Adam DJ, Naik J, Hartshorne T, Bello M, London NJM. The diagnosis and management of 689 chronic leg ulcers in a single-visit assessment clinic. Eur J Vasc Endovasc Surg. 2003 May;25(5):462–8.

5. Overview | Varicose veins: diagnosis and management | Guidance | NICE [Internet]. NICE; 2013 [cited 2023 Jun 2]. Available from: https://www.nice.org.uk/guidance/cg168

6. De Maeseneer MG, Kakkos SK, Aherne T, Baekgaard N, Black S, Blomgren L, et al. Editor's Choice - European Society for Vascular Surgery (ESVS) 2022 Clinical Practice Guidelines on the Management of Chronic Venous Disease of the Lower Limbs. Eur J Vasc Endovasc Surg. 2022 Feb;63(2):184–267.

7. Barwell JR, Davies CE, Deacon J, Harvey K, Minor J, Sassano A, et al. Comparison of surgery and compression with compression alone in chronic venous ulceration (ESCHAR study): randomised controlled trial. Lancet. 2004 Jun 5;363(9424):1854–9.

8. Gohel MS, Heatley F, Liu X, Bradbury A, Bulbulia R, Cullum N, et al. A Randomized Trial of Early Endovenous Ablation in Venous Ulceration. N Engl J Med. 2018 May 31;378(22):2105–14.

9. Brittenden J, Cotton SC, Elders A, Ramsay CR, Norrie J, Burr J, et al. A randomized trial comparing treatments for varicose veins. N Engl J Med. 2014 Sep 25;371(13):1218–27.

For peer review only

For peer review only - http://bmjopen.bmj.com/site/about/guidelines.xhtml

10. Rasmussen LH, Lawaetz M, Bjoern L, Vennits B, Blemings A, Eklof B. Randomized clinical trial comparing endovenous laser ablation, radiofrequency ablation, foam sclerotherapy and surgical stripping for great saphenous varicose veins. Br J Surg. 2011 Aug;98(8):1079–87.

11. Cai PL, Hitchman LH, Mohamed AH, Smith GE, Chetter I, Carradice D. Endovenous ablation for venous leg ulcers. Cochrane Database Syst Rev. 2023 Jul 27;7(7):CD009494.

12. Carradice D, Mekako AI, Mazari F a. K, Samuel N, Hatfield J, Chetter IC. Clinical and technical outcomes from a randomized clinical trial of endovenous laser ablation compared with conventional surgery for great saphenous varicose veins. Br J Surg. 2011 Aug;98(8):1117–23.

13. Carradice D, Mekako AI, Mazari F a. K, Samuel N, Hatfield J, Chetter IC. Randomized clinical trial of endovenous laser ablation compared with conventional surgery for great saphenous varicose veins. Br J Surg. 2011 Apr;98(4):501–10.

14. NHS Digital [Internet]. [cited 2023 Jul 19]. Hospital Admitted Patient Care Activity, 2021-22. Available from: https://digital.nhs.uk/data-and-information/publications/statistical/hospital-admitted-patient-care-activity/2021-22

15. Braekkan SK, Grosse SD, Okoroh EM, Tsai J, Cannegieter SC, Naess IA, et al. Venous thromboembolism and subsequent permanent work-related disability. J Thromb Haemost. 2016 Oct;14(10):1978–87.

16. NHS Digital [Internet]. 2022 [cited 2023 Sep 5]. Deaths from venous thromboembolism (VTE) related events within 90 days post discharge from hospital. Available from: https://digital.nhs.uk/data-and-information/publications/statistical/nhs-outcomes-framework/march-2022

17. Kahn SR, Shapiro S, Wells PS, Rodger MA, Kovacs MJ, Anderson DR, et al. Compression stockings to prevent post-thrombotic syndrome: a randomised placebo-controlled trial. Lancet. 2014 Mar 8;383(9920):880–8.

18. Eklof B, Perrin M, Delis KT, Rutherford RB, Gloviczki P, American Venous Forum, et al. Updated terminology of chronic venous disorders: the VEIN-TERM transatlantic interdisciplinary consensus document. J Vasc Surg. 2009 Feb;49(2):498–501.

19. Noble S, Lewis R, Whithers J, Lewis S, Bennett P. Long-term psychological consequences of symptomatic pulmonary embolism: a qualitative study. BMJ Open. 2014 Apr 1;4(4):e004561.

20. Barco S, Russo M, Vicaut E, Becattini C, Bertoletti L, Beyer-Westendorf J, et al. Incomplete echocardiographic recovery at 6 months predicts long-term sequelae after intermediate-risk pulmonary embolism. A post-hoc analysis of the Pulmonary Embolism Thrombolysis (PEITHO) trial. Clin Res Cardiol. 2019 Jul;108(7):772–8.

21. Bouras G, Burns EM, Howell AM, Bottle A, Athanasiou T, Darzi A. Risk of Post-Discharge Venous Thromboembolism and Associated Mortality in General Surgery: A Population-Based Cohort Study Using Linked Hospital and Primary Care Data in England. PLoS One. 2015;10(12):e0145759.

22. Whittley S, Machin M, Burgess L, Onida S, Carradice D, Davies A. Thromboprophylaxis strategies in patients undergoing endovenous thermal ablation: a UK survey. JVascSocGBIrel [Internet]. 2023 [cited 2023 Nov 16];3. Available from: https://jvsgbi.com/thromboprophylaxis-strategies-in-patients-undergoing-endovenous-thermal-ablation-a-uk-survey/

For peer review only - http://bmjopen.bmj.com/site/about/guidelines.xhtml

23. Boyle E, Reid J, O'Donnell M, Harkin D, Badger S. Thromboprophylaxis for varicose vein procedures - A national survey. Phlebology. 2019 Oct;34(9):598–603.

24. Turner BRH, Machin M, Jasionowska S, Salim S, Onida S, Shalhoub J, et al. Systematic Review and meta-analysis of the Additional Benefit of Pharmacological Thromboprophylaxis for Endovenous Varicose Vein Interventions. Ann Surg. 2022 Sep 15;

25. Recommendations | Venous thromboembolism in over 16s: reducing the risk of hospital-acquired deep vein thrombosis or pulmonary embolism | Guidance | NICE [Internet]. NICE; 2018 [cited 2023 Jun 7]. Available from: https://www.nice.org.uk/guidance/ng89/chapter/Recommendations

26. Dattani N, Shalhoub J, Nandhra S, Lane T, Abu-Own A, Elbasty A, et al. Reducing the risk of venous thromboembolism following superficial endovenous treatment: A UK and Republic of Ireland consensus study. Phlebology. 2020 Oct 1;35(9):706–14.

27. Schulman S, Kearon C, Subcommittee on Control of Anticoagulation of the Scientific and Standardization Committee of the International Society on Thrombosis and Haemostasis. Definition of major bleeding in clinical investigations of antihemostatic medicinal products in non-surgical patients. J Thromb Haemost. 2005 Apr;3(4):692–4.

28. Hwang IK, Shih WJ, De Cani JS. Group sequential designs using a family of type I error probability spending functions. Stat Med. 1990 Dec;9(12):1439–45.

29. Sweetland S, Green J, Liu B, Berrington de González A, Canonico M, Reeves G, et al. Duration and magnitude of the postoperative risk of venous thromboembolism in middle aged women: prospective cohort study. BMJ. 2009 Dec 3;339:b4583.

30. Curtis L, Burns A. Unit Costs of Health and Social Care 2017 [Internet]. Personal Social Services Research Unit, University of Kent; 2017 [cited 2023 Oct 23]. Available from: https://doi.org/10.22024/UniKent/01.02/65559

31. British National Formulary. Tinzaparin sodium, Medicinal forms [Internet]. 2022 [cited 2023 Oct 23]. Available from: https://bnf.nice.org.uk/drugs/tinzaparin-sodium/medicinal-forms/

32. Enden T, Resch S, White C, Wik HS, Kløw NE, Sandset PM. Cost-effectiveness of additional catheter-directed thrombolysis for deep vein thrombosis. J Thromb Haemost. 2013 Jun;11(6):1032–42.

33. NICE. Guide to the methods of technology appraisal (PMG9) [Internet]. NICE; 2023 [cited 2023 Nov 14]. Available from: https://www.nice.org.uk/process/pmg9/chapter/foreword

34. Husereau D, Drummond M, Augustovski F, de Bekker-Grob E, Briggs AH, Carswell C, et al. Consolidated Health Economic Evaluation Reporting Standards 2022 (CHEERS 2022) statement: updated reporting guidance for health economic evaluations. BMC Medicine. 2022 Jan 12;20(1):23.

For peer review only

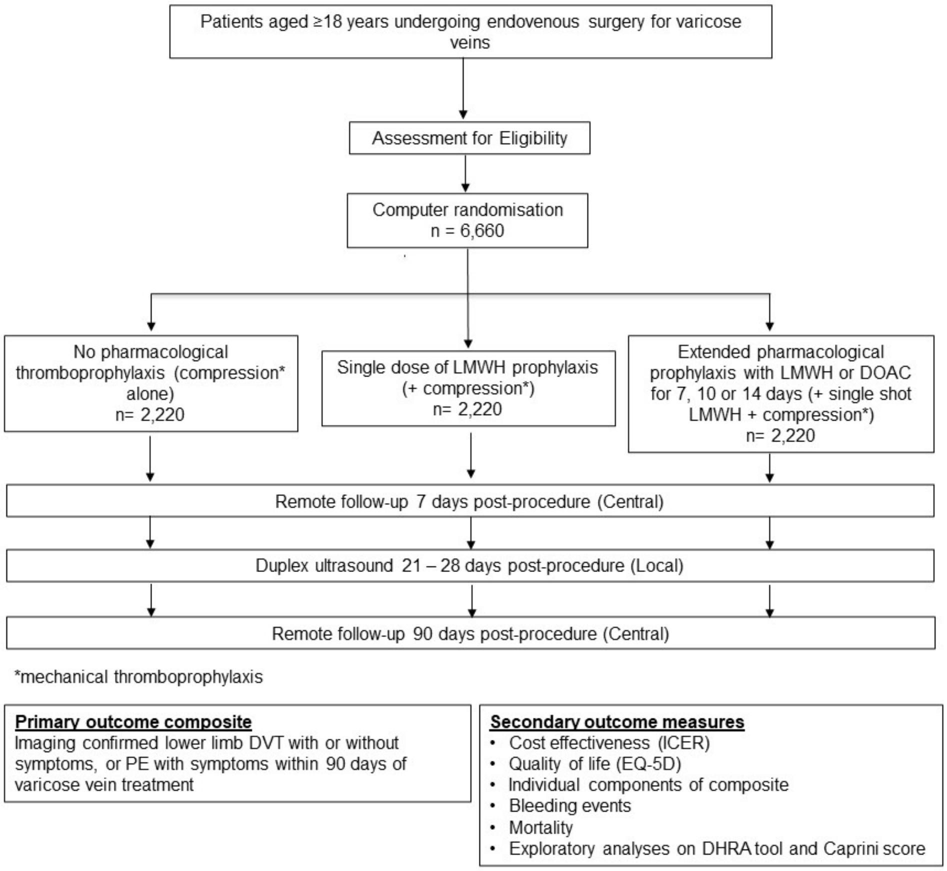

THRIVE study flow chart
Abbreviations: DHRA, Department of Health Risk Assessment; DOAC, direct-acting oral anticoagulant; DVT, deep vein thrombosis; ICER, incremental cost-effectiveness ratio; LMWH, low-molecular weight heparin; VTE, venous thromboembolism.

239x210mm (96 x 96 DPI)

SPIRIT

STANDARD PROTOCOL ITEMS: RECOMMENDATIONS FOR INTERVENTIONAL TRIALS

SPIRIT 2013 Checklist: Recommended items to address in a clinical trial protocol and related documents*

Section/item	Item No	Description
**Administrative information**		
Title	1	Descriptive title identifying the study design, population, interventions, and, if applicable, trial acronym
Trial registration	2a	Trial identifier and registry name. If not yet registered, name of intended registry
	2b	All items from the World Health Organization Trial Registration Data Set
Protocol version	3	Date and version identifier
Funding	4	Sources and types of financial, material, and other support
Roles and responsibilities	5a	Names, affiliations, and roles of protocol contributors
	5b	Name and contact information for the trial sponsor
	5c	Role of study sponsor and funders, if any, in study design; collection, management, analysis, and interpretation of data; writing of the report; and the decision to submit the report for publication, including whether they will have ultimate authority over any of these activities
	5d	Composition, roles, and responsibilities of the coordinating centre, steering committee, endpoint adjudication committee, data management team, and other individuals or groups overseeing the trial, if applicable (see Item 21a for data monitoring committee)
**Introduction**		
Background and rationale	6a	Description of research question and justification for undertaking the trial, including summary of relevant studies (published and unpublished) examining benefits and harms for each intervention
	6b	Explanation for choice of comparators
Objectives	7	Specific objectives or hypotheses
Trial design	8	Description of trial design including type of trial (eg, parallel group, crossover, factorial, single group), allocation ratio, and framework (eg, superiority, equivalence, noninferiority, exploratory)

For peer review only

Methods: Participants, interventions, and outcomes

Study setting	9	Description of study settings (eg, community clinic, academic hospital) and list of countries where data will be collected. Reference to where list of study sites can be obtained
Eligibility criteria	10	Inclusion and exclusion criteria for participants. If applicable, eligibility criteria for study centres and individuals who will perform the interventions (eg, surgeons, psychotherapists)
Interventions	11a	Interventions for each group with sufficient detail to allow replication, including how and when they will be administered
	11b	Criteria for discontinuing or modifying allocated interventions for a given trial participant (eg, drug dose change in response to harms, participant request, or improving/worsening disease)
	11c	Strategies to improve adherence to intervention protocols, and any procedures for monitoring adherence (eg, drug tablet return, laboratory tests)
	11d	Relevant concomitant care and interventions that are permitted or prohibited during the trial
Outcomes	12	Primary, secondary, and other outcomes, including the specific measurement variable (eg, systolic blood pressure), analysis metric (eg, change from baseline, final value, time to event), method of aggregation (eg, median, proportion), and time point for each outcome. Explanation of the clinical relevance of chosen efficacy and harm outcomes is strongly recommended
Participant timeline	13	Time schedule of enrolment, interventions (including any run-ins and washouts), assessments, and visits for participants. A schematic diagram is highly recommended (see Figure)
Sample size	14	Estimated number of participants needed to achieve study objectives and how it was determined, including clinical and statistical assumptions supporting any sample size calculations
Recruitment	15	Strategies for achieving adequate participant enrolment to reach target sample size

Methods: Assignment of interventions (for controlled trials)

Allocation:

Sequence generation	16a	Method of generating the allocation sequence (eg, computer-generated random numbers), and list of any factors for stratification. To reduce predictability of a random sequence, details of any planned restriction (eg, blocking) should be provided in a separate document that is unavailable to those who enrol participants or assign interventions

For peer review only - http://bmjopen.bmj.com/site/about/guidelines.xhtml

Allocation concealment mechanism	16b	Mechanism of implementing the allocation sequence (eg, central telephone; sequentially numbered, opaque, sealed envelopes), describing any steps to conceal the sequence until interventions are assigned
Implementation	16c	Who will generate the allocation sequence, who will enrol participants, and who will assign participants to interventions
Blinding (masking)	17a	Who will be blinded after assignment to interventions (eg, trial participants, care providers, outcome assessors, data analysts), and how
	17b	If blinded, circumstances under which unblinding is permissible, and procedure for revealing a participant's allocated intervention during the trial
**Methods: Data collection, management, and analysis**		
Data collection methods	18a	Plans for assessment and collection of outcome, baseline, and other trial data, including any related processes to promote data quality (eg, duplicate measurements, training of assessors) and a description of study instruments (eg, questionnaires, laboratory tests) along with their reliability and validity, if known. Reference to where data collection forms can be found, if not in the protocol
	18b	Plans to promote participant retention and complete follow-up, including list of any outcome data to be collected for participants who discontinue or deviate from intervention protocols
Data management	19	Plans for data entry, coding, security, and storage, including any related processes to promote data quality (eg, double data entry; range checks for data values). Reference to where details of data management procedures can be found, if not in the protocol
Statistical methods	20a	Statistical methods for analysing primary and secondary outcomes. Reference to where other details of the statistical analysis plan can be found, if not in the protocol
	20b	Methods for any additional analyses (eg, subgroup and adjusted analyses)
	20c	Definition of analysis population relating to protocol non-adherence (eg, as randomised analysis), and any statistical methods to handle missing data (eg, multiple imputation)
**Methods: Monitoring**		
Data monitoring	21a	Composition of data monitoring committee (DMC); summary of its role and reporting structure; statement of whether it is independent from the sponsor and competing interests; and reference to where further details about its charter can be found, if not in the protocol. Alternatively, an explanation of why a DMC is not needed

	21b	Description of any interim analyses and stopping guidelines, including who will have access to these interim results and make the final decision to terminate the trial
Harms	22	Plans for collecting, assessing, reporting, and managing solicited and spontaneously reported adverse events and other unintended effects of trial interventions or trial conduct
Auditing	23	Frequency and procedures for auditing trial conduct, if any, and whether the process will be independent from investigators and the sponsor
**Ethics and dissemination**		
Research ethics approval	24	Plans for seeking research ethics committee/institutional review board (REC/IRB) approval
Protocol amendments	25	Plans for communicating important protocol modifications (eg, changes to eligibility criteria, outcomes, analyses) to relevant parties (eg, investigators, REC/IRBs, trial participants, trial registries, journals, regulators)
Consent or assent	26a	Who will obtain informed consent or assent from potential trial participants or authorised surrogates, and how (see Item 32)
	26b	Additional consent provisions for collection and use of participant data and biological specimens in ancillary studies, if applicable
Confidentiality	27	How personal information about potential and enrolled participants will be collected, shared, and maintained in order to protect confidentiality before, during, and after the trial
Declaration of interests	28	Financial and other competing interests for principal investigators for the overall trial and each study site
Access to data	29	Statement of who will have access to the final trial dataset, and disclosure of contractual agreements that limit such access for investigators
Ancillary and post-trial care	30	Provisions, if any, for ancillary and post-trial care, and for compensation to those who suffer harm from trial participation
Dissemination policy	31a	Plans for investigators and sponsor to communicate trial results to participants, healthcare professionals, the public, and other relevant groups (eg, via publication, reporting in results databases, or other data sharing arrangements), including any publication restrictions
	31b	Authorship eligibility guidelines and any intended use of professional writers
	31c	Plans, if any, for granting public access to the full protocol, participant-level dataset, and statistical code

For peer review only - http://bmjopen.bmj.com/site/about/guidelines.xhtml

Appendices

Informed consent materials	32	Model consent form and other related documentation given to participants and authorised surrogates
Biological specimens	33	Plans for collection, laboratory evaluation, and storage of biological specimens for genetic or molecular analysis in the current trial and for future use in ancillary studies, if applicable

*It is strongly recommended that this checklist be read in conjunction with the SPIRIT 2013 Explanation & Elaboration for important clarification on the items. Amendments to the protocol should be tracked and dated. The SPIRIT checklist is copyrighted by the SPIRIT Group under the Creative Commons "Attribution-NonCommercial-NoDerivs 3.0 Unported" license.

For peer review only

BMJ Open

Evaluating pharmacological THRomboprophylaxis in Individuals undergoing superficial endoVEnous treatment across NHS and private clinics in the UK: a multi-centre, assessor blind, randomised controlled trial – THRIVE trial

Journal:	*BMJ Open*
Manuscript ID	bmjopen-2023-083488.R1
Article Type:	Protocol
Date Submitted by the Author:	16-Jan-2024
Complete List of Authors:	Machin, Matthew; Imperial College London, Section of Vascular Surgery, Department of Surgery and Cancer, Charing Cross Hospital; Imperial College Healthcare NHS Trust, Imperial Vascular Unit, St Mary's Hospital<br>Whittley, Sarah; Imperial College London, Section of Vascular Surgery, Department of Surgery and Cancer, Charing Cross Hospital<br>Norrie, John; The University of Edinburgh Usher Institute of Population Health Sciences and Informatics, Edinburgh Clinical Trials Unit, University of Edinburgh, Level 2, NINE Edinburgh BioQuarter<br>Burgess, Laura; Imperial College London, Section of Vascular Surgery, Department of Surgery and Cancer, Charing Cross Hospital<br>Hunt, Beverley; Guy's & St Thomas' Foundation Trust, Thrombosis & Haemophilia Centre, Westminster Bridge Road<br>Bolton, Layla; Imperial College Healthcare NHS Trust, Imperial Vascular Unit, St Mary's Hospital<br>Shalhoub, Joseph; Imperial College London, Section of Vascular Surgery, Department of Surgery and Cancer, Charing Cross Hospital; Imperial College Healthcare NHS Trust, Imperial Vascular Unit, St Mary's Hospital<br>Everington, Tamara; Hampshire Hospitals NHS Foundation Trust, Aldermaston Road, Basingstoke<br>Gohel, Manjit; Cambridge University Hospitals NHS Foundation Trust, Cambridge Vascular Unit, Addenbrookes Hospital, Hills Road<br>Whiteley, Mark S; University of Surrey<br>Rogers, Steven; The University of Manchester, Division of Cardiovascular Sciences, School of Medical Sciences, Faculty of Biology, Medicine and Health, Manchester Academic Health Science Centre; Manchester University NHS Foundation Trust, Manchester Academic Vascular Research and Innovation Centre (MAVRIC), Oxford Road<br>Onida, Sarah; Imperial College London, Academic Section of Vascular Surgery Department of Surgery & Cancer Imperial College London<br>Turner, Benedict; Imperial College London, Section of Vascular Surgery, Department of Surgery and Cancer, Charing Cross Hospital<br>Nandhra, Sandip; Newcastle University, Population Health Sciences Institute, Newcastle Upon Tyne<br>Lawton, Rebecca; Imperial College London, Academic Section of Vascular Surgery<br>Stephens-Boal, Annya; Imperial College London, Department of Surgery & Cancer<br>Singer, Carolyn; Charing Cross Hospital, Department of Surgery and

	Cancer<br>Dunbar, Joanne; Charing Cross Hospital, Department of Surgery and Cancer<br>Carradice, Daniel; Hull York Medical School; Hull University Teaching Hospitals NHS Trust<br>Davies, AH; Charing Cross Hospital, Department of Surgery and Cancer; St Mary’s Hospital
**Primary Subject Heading**:	Surgery
Secondary Subject Heading:	Cardiovascular medicine
Keywords:	VASCULAR SURGERY, Anticoagulation < HAEMATOLOGY, Thromboembolism < CARDIOLOGY

For peer review only - http://bmjopen.bmj.com/site/about/guidelines.xhtml

Title: Evaluating pharmacological THRomboprophylaxis in Individuals undergoing superficial endoVEnous treatment across NHS and private clinics in the UK: a multi-centre, assessor blind, randomised controlled trial – THRIVE trial

Current protocol version: V3.0, 10-Oct-2023

Authors: Matthew Machin[1,2], Sarah Whittley[1], John Norrie[3], Laura Burgess[1], Beverley J Hunt[4], Layla Bolton-Saghdaoui[2], Joseph Shalhoub[1,2], Tamara Everington[5], Manjit Gohel[6], Mark Whiteley[7], Steven Rogers[8,9], Sarah Onida[1,2], Benedict Turner[1], Sandip Nandhra[10], Rebecca Lawton[1], Annya Stephens-Boal[1], Carolyn Singer[1], Joanne Dunbar[1], Daniel Carradice[11], Alun Huw Davies[1,2.]

[1] Section of Vascular Surgery, Department of Surgery and Cancer, Imperial College London, Charing Cross Hospital, London W6 8RF, United Kingdom.

[2] Imperial Vascular Unit, Imperial College Healthcare NHS Trust, St Mary's Hospital, London W2 1NY, United Kingdom.

[3] Usher Institute of Population Health Sciences and Informatics, Edinburgh Clinical Trials Unit, University of Edinburgh, Level 2, NINE Edinburgh BioQuarter, 9 Little France Road, Edinburgh, EH16 4UX, United Kingdom

[4] Thrombosis & Haemophilia Centre, Guy's and St Thomas' NHS Foundation Trust, Westminster Bridge Road, London, SE1 7EH, United Kingdom.

[5] Hampshire Hospitals NHS Foundation Trust, Aldermaston Road, Basingstoke, Hampshire, RG24 9NA, United Kingdom.

For peer review only

[6] Cambridge Vascular Unit, Addenbrookes Hospital, Cambridge University Hospitals NHS Foundation Trust, Hills Road, Cambridge, CB2 0QQ, United Kingdom.

[7] The Whiteley Clinic, 1 Chapel Place, London, W1G 0BG, United Kingdom

[8] Division of Cardiovascular Sciences, School of Medical Sciences, Faculty of Biology, Medicine and Health, University of Manchester, Manchester Academic Health Science Centre, Manchester, M13 9PL, United Kingdom.

[9]Manchester Academic Vascular Research and Innovation Centre (MAVRIC), Manchester University NHS Foundation Trust, Oxford Road, Manchester, M13 9WL, United Kingdom

[10] Population Health Sciences Institute, Newcastle University, Newcastle Upon Tyne, NEW1 7RU, United Kingdom.

[11] Academic Vascular Surgical Unit, Hull York Medical School, Allam Diabetes Centre, Hull Royal Infirmary, Anlaby Road, Hull, HU3 2JZ, United Kingdom

Professor Alun Davies and Mr Daniel Carradice should be considered joint senior author.

Corresponding author: Professor Alun H. Davies, Professor of Vascular Surgery, Section of Vascular Surgery, Department of Surgery and Cancer, Imperial College London, Charing Cross Hospital, London W6 8RF, United Kingdom. Email: a.h.davies@imperial.ac.uk.

For peer review only

ABSTRACT

Introduction

Endovenous therapy is the first-choice management for symptomatic varicose veins in NICE guidelines, with 56-70,000 procedures performed annually in the UK. Venous thromboembolism (VTE), including deep vein thrombosis (DVT) and pulmonary embolism (PE) is a known complication of endovenous therapy, occurring at a rate of up to 3.4%. Despite 73% of UK practitioners administering pharmacological thromboprophylaxis to reduce VTE, no high-quality evidence supporting this practice exists. Pharmacological thromboprophylaxis may have clinical and cost benefit in preventing VTE, however, further evidence is needed. This study aims to establish whether when endovenous therapy is undertaken: a single dose or course of pharmacological thromboprophylaxis alters the risk of VTE; pharmacological thromboprophylaxis is associated with an increased rate of bleeding events; pharmacological prophylaxis is cost effective.

Methods and analysis

A multi-centre, assessor-blind, randomised controlled trial (RCT) will recruit 6660 participants from forty NHS and private sites across the UK. Participants will be randomised to intervention (single dose or extended course of pharmacological thromboprophylaxis plus compression) or control (compression alone). Participants will undergo a lower limb venous duplex ultrasound scan at 21-28 days post-procedure to identify asymptomatic DVT. The duplex scan will be conducted locally by blinded assessors. Participants will be contacted remotely for follow-up at 7-days and 90-days post-procedure. The primary outcome is imaging confirmed lower limb DVT with or without symptoms, or PE with symptoms within 90 days of treatment. The main analysis will be according to the intention-to-treat principle and will compare the rates of VTE at 90 days, using a repeated measures analysis

For peer review only - http://bmjopen.bmj.com/site/about/guidelines.xhtml

of variance (ANOVA), adjusting for any pre-specified strongly prognostic baseline covariates using a mixed effects logistic regression.

Ethics and dissemination

Ethical approval was granted by Brent Research Ethics Committee (22/LO/0261). Results will be disseminated in a peer-reviewed journal and presented at national and international conferences.

Trial registration number

ISRCTN18501431

KEYWORDS

Venous thromboembolism, deep vein thrombosis, pulmonary embolism, superficial endovenous treatment, thromboprophylaxis, low molecular weight heparin, direct-acting oral anticoagulants.

ARTICLE SUMMARY

Strengths and limitations of this study

- The primary outcome holds clinical significance.
- Using VTE prophylaxis may be associated with adverse clinical outcomes, increased risks and may not be cost-effective.

For peer review only - http://bmjopen.bmj.com/site/about/guidelines.xhtml

- Due to the 90 day follow-up period, long-term efficacy of the intervention will not be assessed.

Word count: 4,000

For peer review only

For peer review only - http://bmjopen.bmj.com/site/about/guidelines.xhtml

INTRODUCTION

Varicose veins, also known as superficial refluxing veins, affect up to 45% of the UK population (1). Varicose veins not only reduce physical and mental health-related quality of life, but also contribute significantly to chronic venous disease, which is responsible for over half of all cases of leg ulcers (2–4). Superficial endovenous treatment (SET), offers a minimally invasive approach that can conveniently be performed in an outpatient setting (5). Endovenous surgery stands as the recommended first-choice management for symptomatic varicose veins, in line with NICE guidelines [CG168] (5). This recommendation aligns with the European Society for Vascular Surgery Clinical Practice Guidelines, providing a level I recommendation for endovenous techniques (6). SET not only improves quality of life and facilitates venous ulcer healing, but also offers cost savings to healthcare providers (7–13). Annually, approximately 30,000 endovenous varicose vein procedures are carried out within the NHS, a number estimated to reach 68,800 with full adherence to NICE guidelines (14). Additionally, an estimated 30,000 to 40,000 procedures are undertaken annually in the private sector.

Despite its efficacy, SET is associated with thrombotic complications, presenting occurrences of venous thromboembolism (VTE) and endothermal heat-induced thrombosis (EHIT) at rates as high as 3.4%. VTE, encompassing deep vein thrombosis (DVT) and pulmonary embolism (PE), is a significant cause of disability and subsequent societal economic consequences (15). Hospital-acquired thrombosis (HAT), which is defined as the development of VTE within 90 days of a hospital episode, significantly contributes to morbidity and mortality, with statistics indicating a rate of 57 deaths per 100,000 admissions within the NHS (16). Complications following a DVT are substantial, with up to 50% of patients developing post thrombotic syndrome (PTS), characterised by chronic leg pain, oedema and skin changes (17,18). Additionally, PE is associated with lifelong functional and psychological repercussions, ultimately posing a risk of death during the index event (19,20). EHIT,

For peer review only - http://bmjopen.bmj.com/site/about/guidelines.xhtml

For peer review only

encompassing any thrombus forming within 4 weeks of endovenous ablation and extending from the treated vein toward or into a deep vein, is categorised into four classes. EHIT classes 3 – 4 involve significant thrombus extension into and encompassing the deep vein, often necessitating treatment similar to that for DVT (9,10). SET presents a unique VTE risk compared to other short-stay surgical procedures, exhibiting a VTE rate comparable to that observed in major joint surgeries (16). Comparable day-case surgical procedures such as inguinal hernia repair or laparoscopic cholecystectomy exhibit a notably lower VTE rate of 0.3% (21).

In attempting to reduce the risk of VTE, UK clinicians exhibit varying approaches: 52% routinely prescribe a single dose of low-molecular weight heparin (LMWH) and 15% routinely prescribe extended pharmacological thromboprophylaxis with either LMWH or a direct-acting oral anticoagulant (DOAC), while 33% do not prescribe any pharmacological thromboprophylaxis (22). These practices align with findings from a 2019 national survey of vascular surgeons in Ireland, indicating that 73% of practitioners routinely prescribe pharmacological thromboprophylaxis for SET, utilising either a single dose of LMWH or extended prophylaxis, while 27% of practitioners do not prescribe any form of pharmacological thromboprophylaxis (23). Contradicting NICE guidelines, the routine use of pharmacological thromboprophylaxis for SET has become prevailing practice, despite having no supportive evidence base. A systematic review and meta-analysis published in 2022 failed to identify any high-quality evidence to support current pharmacological thromboprophylaxis strategies in this group of patients (24). However, the study suggested there is a significant reduction in the rate of DVT with additional pharmacological thromboprophylaxis (1.09% versus 3.20% for pharmacological thromboprophylaxis versus compression alone). International and national guidelines reflect the paucity of evidence in this area, with the European Society of Vascular Surgery guidelines providing a (IIa B) recommendation for consideration of individualised thromboprophylaxis strategies (6). This is exacerbated by NICE NG89 recommending that "prophylaxis is generally not needed for people undergoing varicose vein surgery" if their VTE risk assessment deems them low-risk (25).

For peer review only - http://bmjopen.bmj.com/site/about/guidelines.xhtml

Clinicians also lack confidence in current risk assessment tools (RATs) for patients undergoing SET (15). Despite the utilisation of RATs such as the Department of Health Risk Assessment (DHRA) Tool in the UK and the Caprini RAT in Europe and the US, none have undergone validation for varicose vein procedures. Consequently, varicose vein intervention-specific RATs have emerged, yet these, too, lack validation. Thus, there is currently no consensus on which risk factors provide clinical indication for pharmacological thromboprophylaxis or confer high-risk for VTE (26).

Pharmacological thromboprophylaxis presents potential clinical and cost benefits in VTE prevention, however grade A evidence is required to either support or refute this practice. Furthermore, as current RATs have not been validated in this patient group, unbiased prospective evidence will help guide risk-stratifying patients in the future. This research question also aligns with the James Lind Alliance priority setting for venous disease, further underlining the need for this trial.

Objectives

The aims of this study are to establish whether in patients undergoing SET:

1) A single dose of pharmacological thromboprophylaxis decreases the risk of VTE
2) An extended course of pharmacological thromboprophylaxis decreases the risk of VTE
3) Pharmacological thromboprophylaxis is associated with an increased rate of bleeding events
4) Providing pharmacological prophylaxis is cost-effective
5) Any pharmacological thromboprophylaxis affects the rate of VTE

For peer review only - http://bmjopen.bmj.com/site/about/guidelines.xhtml

METHODS AND ANALYSIS

Trial design

This is a multi-centre, assessor-blind randomised controlled trial with a superiority comparison. The primary outcome is assessed blindly.

Study setting

This trial will take place in NHS hospitals and private clinics delivering endovenous varicose vein procedures under local anaesthesia. Recruitment centres will need to have a pre-existing practice prior to the trial to prevent any learning curve effects.

Eligibility criteria

Inclusion criteria are: adult patients (>18 years) scheduled to undergo endovenous intervention of truncal varicose veins under local anaesthesia. Treatment technologies include radiofrequency, laser, mechanochemical, foam sclerotherapy and cyanoacrylate glue.

Exclusion criteria are: clinical indication for therapeutic anticoagulation (e.g. atrial fibrillation, previous personal or first-degree relative history of VTE, thrombophilia). Also female patients of childbearing potential with a positive pregnancy test; those with a history of allergy to heparins or DOACs; a history of heparin-induced thrombocytopenia in the last year; inherited and acquired bleeding disorders; evidence of active bleeding; concomitant major health problems such as active cancer and chronic renal and/or liver impairment; known thrombocytopenia (platelets known to be less than 50 x 10^9/l); surgery or major trauma in the previous 90 days; recent ischaemic stroke in the previous 90 days; inability to provide consent.

For peer review only - http://bmjopen.bmj.com/site/about/guidelines.xhtml

Interventions

There are currently three thromboprophylaxis strategies used across the UK, with the trial arms of this application mirroring these practices (23). Intervention arms will consist of a single prophylactic dose of LMWH (e.g. dalteparin sodium, tinzaparin sodium, enoxaparin sodium) plus compression as per local practice (e.g. stockings, bandages, wraps, pads), and a single dose of LMWH plus extended thromboprophylaxis with LMWH or a DOAC (e.g. rivaroxaban, apixaban, dabigatran etexilate) plus compression. The choice between LMWH or DOAC for the extended thromboprophylaxis arm will be site-specific and dependent on local practice. The duration of this must be at least 7 days, but can be in line with local practice i.e., between 7 and 14 days in duration. The control arm will consist of compression (bandages, stockings, wraps or padding) as per local practice alone. The participant flow in the trial is displayed in *Figure 1*.

Primary outcome

Imaging confirmed lower limb DVT with or without symptoms, or PE with symptoms within 90 days of varicose vein treatment.

Secondary outcomes

The secondary outcomes include:

1) Individual components of the composite outcome
2) Comparisons of quality of life at 7- and 90-days post-procedure using the EQ-5D
3) Mortality rates in each group
4) Cost-effectiveness of providing pharmacological thromboprophylaxis

For peer review only - http://bmjopen.bmj.com/site/about/guidelines.xhtml

5) Exploratory analyses to assess how well the DHRA tool and Caprini score predict outcome

Safety outcomes

Safety monitoring includes any bleeding event. Major bleeding is defined as per the International Society on Thrombosis and Haemostasis standardised definition (27), which includes:

1) Bleeding into a critical organ
2) Bleeding into a surgical site requiring re-operation
3) Bleeding that leads to presentation to acute service

Sample size and study duration

The most comprehensive evidence from 52 studies suggests that the rate of VTE (encompassing DVT, PE, EHIT) post-endovenous great saphenous vein interventions is 1.7% (1). However, this analysis did not investigate treatment effects. To address this, we conducted an analysis of 229 study arms, pooling data from 480,581 participants undergoing endovenous interventions. Our approach involved subgroup analyses, comparing those receiving pharmacological thromboprophylaxis and compression versus compression alone, and distinguishing between asymptomatic screen-detected and symptomatic VTE (24).

For individuals receiving pharmacological thromboprophylaxis (either single dose of LMWH or extended duration), rates of DVT, PE, and EHIT 3 - 4 were 0.521%, 0.216%, and 0.354%, respectively, resulting in a maximum summary VTE rate of 1.09%. Those receiving compression alone showed rates of 2.264% for DVT, 0.058% for PE, and 0.878% for EHIT 3- 4, resulting in a maximum summary VTE rate of 3.20%. Rates of EHIT and DVT are distinct in this calculation, thus the impact of double counting contributing events will be negligible. In the subset data from RCT arms alone, rates of DVT were similar. When interpreting these figures in the context of the wider literature, balancing this with

For peer review only - http://bmjopen.bmj.com/site/about/guidelines.xhtml

confounding by indication, and acknowledging that VTE rate will likely be lower in the compression alone arm due to the exclusion of individuals at the highest risk of VTE, we anticipate that the true value lies nearer to 1.0% in the pharmacological thromboprophylaxis arm and 2.7% in the compression alone arm.

At 90% power and 2.5% alpha (to approximately control overall alpha to 5%, with two active drugs being compared with a common control), the study could detect a significant change of 1.7% in 90-day VTE. This base case would require 1554 participants per group. Allowing for 10% crossover (which can only be control patients, compression only, receiving pharmacological thromboprophylaxis, since everyone receives compression) increases this to 1919 per group. Allowing for a single interim analysis at half time (50% randomised with 90 day follow up) for early stopping, for either futility or overwhelming evidence of efficacy analysis, inflates this to 1998 per group, under a two-sided, asymmetric, group sequential design implementing the non-binding Hwang-Shih-DeCani spending function [lower bound (futility) with gamma-2; upper bound (efficacy) with gamma= -4] (28). If we then allow 10% for loss to follow up, the total sample, randomised 1:1:1 between all groups, becomes 6660 (or 2220 per group).

We will more accurately estimate the required sample size by simulation in a sample size re-estimation step at around 20% mature data, using Dunnett's 3-arm design with a common control, with correction, but for simplicity retaining the 1:1:1 equal randomisation; and inputting the observed missing data proportion at that stage.

Interim analysis

There will be a formal interim analysis with the possibility of stopping early for futility (no prospect of a clinically meaningful treatment effect), at the point of 50% mature primary outcome data. Full details of the stopping boundaries and analysis will be detailed in a Statistical Analysis Plan (SAP),

For peer review only - http://bmjopen.bmj.com/site/about/guidelines.xhtml

which the independent Data Monitoring Committee (iDMC) will approve prior to seeing unblinded data. The unblinded statistician will have no other role in the study while it is ongoing. The stopping rules are statistically non-binding. The iDMC may recommend early stopping of the study if the boundaries are crossed. They would make a recommendation to the independent Trial Steering Committee (TSC) who may or may not endorse that recommendation. The trial may stop at any time for safety if there is an excess of events in the intervention groups that is considered to generate avoidable harm. This decision would not be based on any statistical criterion and would be taken by the iDMC, then endorsed by the TSC.

Recruitment and randomisation

Adults scheduled to undergo SET will be pre-screened by a member of the direct care team and invited to discuss the trial with a member of the research team. Informed consent will be obtained from each participant prior to participation in the trial, following adequate explanation of the aims, methods, anticipated benefits, and potential hazards of the trial. The individual obtaining consent will be a registered healthcare professional and will have been delegated this duty by the Principal Investigator (PI) on the delegation log. Participants may provide their consent to participate in the trial electronically or in written form. Participants will initial each statement on the consent form to confirm agreement. A copy of the consent form will be provided to the participant for their records. There is no defined timeframe between initial consent and the baseline visit, however confirmation that the participant's consent is still valid prior to randomisation will be required in the baseline case report form (CRF).

Participants (n = 6,660) will undergo 1:1:1 web-based randomisation to one of three thromboprophylaxis strategies prior to undergoing SET. Randomisation will be conducted by an automated system linked to the Research Electronic Data Capture (REDCap) database setup via the Study Data Centre at the Edinburgh Clinical Trials Unit (ECTU), University of Edinburgh (a fully

For peer review only - http://bmjopen.bmj.com/site/about/guidelines.xhtml

registered UKCRC Clinical Trials Unit, registration number 15). Participants will be assigned a study identification number by REDCap in sequential numerical order. Recruitment will commence on 01 January 2024 for 27 months. The study will close on 31 December 2026.

Blinding

Clinicians and participants will be aware of their treatment allocation. Assessors, being those who perform the venous duplex ultrasound scan and those responsible for collecting follow-up data at 7- and 90- days post-procedure, will be blinded to the treatment allocation.

Follow-up periods

Participants will undergo a lower limb venous duplex ultrasound scan at 21 – 28 days post-intervention to identify asymptomatic DVT. This is timed to capture the peak onset of events which is at 3 weeks (29). Participants will be further followed up remotely by telephone, online or short message service (SMS) at 7- and 90-days with an expected VTE capture >95% (29). Longer term follow-up may be considered with award from a subsequent project grant to assess long-term efficacy of the intervention, hence contact at a later point will be included in the consenting process.

Data collection and confidentiality

Participant data will be entered into the REDCap database by the local research teams. Source data stored at study centres will be archived locally as per local study operating procedures. Data and all appropriate documentation will be stored for a minimum of 10 years after the completion of the study, including the follow-up period. Details of procedures for CRF completion will be provided in a separate study manual. A formal Data Management Plan will be constructed to describe the

For peer review only - http://bmjopen.bmj.com/site/about/guidelines.xhtml

procedures involved in the data management activities and processes for the study so that it is managed and maintained in accordance with the Good Clinical Practice (GCP) guidelines, local Research and Governance Integrity Team (RGIT) standard operating procedures, appropriate regulatory requirements, and the study protocol.

Statistical analyses

All statistical analyses will be governed by a comprehensive SAP, written by the study statistician, and agreed by the TSC and iDMC before any unblinded data is seen. The main analysis will be according to the intention-to-treat principle where all consented participants will be included in the analysis retained in the group to which they were allocated (i.e., "as randomised") and for whom outcome data are available. All results will be presented as point estimates, confidence intervals at the appropriate level and associated p-values. Absolute measures of effect will be presented alongside relative measures. The primary analysis will compare the incidence of VTE at 90 days, using a repeated measures analysis of variance (ANOVA), adjusting for any pre-specified strongly prognostic baseline covariates using a mixed effects logistic regression. Study site will be included in the model as a random effect and pre-specified baseline covariates strongly related to outcome will be included to adjust the estimated treatment effect. The findings will be assessed for robustness against any missing data, first using multiple imputation assuming this data is missing at random and, if appropriate and the data permits, further sensitivity analyses will be attempted under any plausible missing data mechanisms not missing at random. Secondary outcomes will be analysed in a similar fashion with generalised linear models appropriate to the distribution of the outcome. Safety data will be summarised descriptively.

For peer review only - http://bmjopen.bmj.com/site/about/guidelines.xhtml

Internal pilot

There will be an internal pilot to assess feasibility of recruitment over nine months of recruitment to the trial, in which we will start recruiting from the (minimum) 40 centres. Site setup will be staggered over nine months, i.e., five centres per month. The target number of participants by the end of the 9-month internal pilot is 1450 participants. For the internal pilot, we will use stop-go criteria based on a Green-Amber-Red statistical approach (*Table 1*).

**Progression criteria**	**Red**	**Amber**	**Green**
% Threshold	<10.9%	10.9 – 20.9%	≥21%
Trial recruitment (of eligible participants)	<15%	15 – 17.6%	≥17.6%
Recruitment rate / site / month	<5	5 – 7.1	≥7.2
Number of centres opened	<25	25 – 40	≥40
Total number of participants recruited	<725	725 – 1449	≥1450

Table 1. Internal pilot of feasibility assessment at 9 months.

Cost-effectiveness analysis

Two health economic analyses will be conducted, and a separate Health Economics Analysis Plan (HEAP) will be developed by the Health Economist detailing the proposed analyses. The main analyses will be performed from the perspective of the NHS and Personal Social Services, with secondary analyses from a societal perspective.

A within-trial analysis will compare the two pharmacological thromboprophylaxis strategies to compression alone over the 90 days of the study. Resource use items associated with treatments in primary and secondary care will be collected using case notes and self-completed patient diaries, and costed using manufacturer list prices, previous literature, and national reference costs. Days off work and normal activities and other patient-related costs will be collected for a secondary analysis. EQ-5D will be collected at baseline and follow up, analysed using the NICE approved tariff.

For peer review only

For peer review only - http://bmjopen.bmj.com/site/about/guidelines.xhtml

If the trial indicates that pharmacological thromboprophylaxis could be an effective therapy, a Markov (state–transition) decision model will be constructed to compare the cost effectiveness of the two pharmacological thromboprophylaxis strategies and compression alone over a longer time horizon. The time horizon of the model will be 2-years, allowing extrapolation of sequelae of VTE events (such as PTS) over the longer term to quantify the impact of VTE on patient health via quality adjusted life years (QALYs) and resource use. A preliminary model has been constructed based on published literature to identify the key variables that would need to be collected during the clinical study, and to estimate the number needed to treat (NNT) to avoid one VTE, above which pharmacological thromboprophylaxis would not be considered cost-effective at NICE thresholds. This model conservatively assumes 30% of patients with VTE develop PTS, with 3% of those having severe PTS.

The minimum cost of purchasing 10-days of thromboprophylaxis and providing allocated time for administration training equates to ~£63.13 (30,31). Our model assumes cost of treatment of VTE, non-severe and severe PTS, as £451, £872 and £1,547 respectively and estimates of the respective utility decrement associated with symptomatic VTE and PTS are 0.8628, 0.7745 and 0.6752 respectively (32). Different durations of thromboprophylaxis will be modelled. These estimates will be reviewed at the time of the cost-effectiveness analysis, and any changes to these estimates will be updated.

Utilising a 2-year time horizon, incremental cost effectiveness ratio of pharmacological thromboprophylaxis in comparison to no pharmacological therapy would be £13,339 per QALY if the NNT were 59 participants (1/0.017). For pharmacological thromboprophylaxis to be cost effective at a NICE willingness to pay threshold of £20,000 per QALY, the NNT would need to be below 80.

Main analyses will be undertaken from perspective of the NHS and Personal Social Services, with secondary analyses from a societal perspective. Health economic analysis will be conducted according to NICE reference case and CHEERS guidelines, including sensitivity analyses and

For peer review only - http://bmjopen.bmj.com/site/about/guidelines.xhtml

probabilistic sensitivity analyses (33,34). Results will be presented as estimates of mean incremental costs, effects, and incremental cost per QALY.

Data monitoring, safety and quality control

An independent TSC and iDMC have been convened. The role of the TSC is to provide overall supervision of trial conduct and progress, and ensure the study adheres to GCP principles. The role of the iDMC is to oversee the safety of the trial participants, and the iDMC will be the only oversight committee that sees unblinded data as the trial progresses, which they will keep in strict confidence. Details of membership, responsibilities and frequency of meetings for the TSC and iDMC have been defined in separate Charters.

The Trial Management Group will oversee trial progress. All adverse events (AEs) will be collected and recorded on the REDCap database. Causality and relatedness will be assigned by the local PI or other appropriately trained and delegated individual. Research sites will inform the Coordinating Centre of all serious adverse events (SAEs) within 24 hours of knowledge of the event.

A study-specific risk assessment has been prepared in preparation for the study by the Trial Manager and study sponsor, which will be updated as required during the course of the trial. The frequency, type and intensity of monitoring visits will be detailed in a separate Data Monitoring Plan (DMP). The DMP will also detail the procedures for completion and sign-off of monitoring reports. In the event of a request for a trial site inspection by any regulatory authority, the study coordinating team must be notified as soon as possible. Participating investigators must agree to allow trial related monitoring, including audits, Research Ethics Committee (REC) review and regulatory inspections, by providing access to source data and other trial related documentation as required. Participant consent for this must be obtained as part of the informed consent process for the trial. The trial

For peer review only

coordinating centre will centrally review eCRF data for errors and missing key data points on an ongoing basis.

Quality Control will be performed according to Imperial College internal procedures. The study may be audited by a Quality Assurance representative of the Sponsor. All necessary data and documents will be made available for inspection. The study may be subject to inspection and audit by regulatory bodies to ensure adherence to GCP and the UK Policy Framework for Health and Social Care Research.

Patient and public involvement

Regular sustained patient and public involvement (PPI) has been undertaken to design and guide the study. Successful focus groups and interviews were held with patient representatives longitudinally throughout the trial development to gain insights into patients' lived experiences and gather feedback on potential trial designs. Online surveys were also conducted with the wider venous community to inform the Plain English Summary. The patient-facing documentation has been designed in collaboration with three patient representatives. Two patient advisers have agreed to sit on the TSC, as well as review updated patient-facing documentation and assist with the dissemination of study results. An online survey of key stakeholders was also undertaken at the UK's largest national conference to ascertain acceptability among clinicians of the proposed trial design.

ETHICS AND DISSEMINATION

For peer review only - http://bmjopen.bmj.com/site/about/guidelines.xhtml

The Study Coordination Centre has obtained approval from the London Brent REC (23/LO/0261). Protocol amendments will be submitted to the sponsor for review before applying for approval from the REC, Health Research Authority (HRA) and Medicines and Healthcare Products Regulatory Agency (MHRA) and updating the ICRCTN record accordingly. The study must also receive confirmation of capacity and capability from each participating NHS Trust before accepting any research activity is carried out. The study will be conducted in accordance with the recommendations for physicians involved in research on human subjects adopted by the 18th World Medical Assembly, Helsinki 1964 and later revisions. Study findings will guide international clinical practice and stimulate key updates to international guidelines. Results will be published in high-impact journals alongside presentation at national and international vascular and haematology societies.

Funding statement

This work is supported by the National Institute for Health and Care Research (NIHR) Health Technology Assessment (HTA) programme (NIHR152877). The views expressed are those of the author(s) and not necessarily those of the NIHR or the Department of Health and Social Care.

Current study status

The study is currently in the recruitment phase.

Trial sponsor

Imperial College London is the main sponsor for this study. Delegated responsibilities are assigned to the NHS Trusts/Health Boards and non-NHS sites taking part in this study.

For peer review only - http://bmjopen.bmj.com/site/about/guidelines.xhtml

Availability of data and materials

Data will be made available on reasonable request.

Competing interests

The authors have no competing interests to declare.

Author contributions

The author contributions were as follows: Conception (AHD, DC and MM); Design of the work (AHD, DC, MM, SW, JN, LB, BJH, LB-S, JS, TE, MG, MW, SR, SO, BT, SN, RL, AS-B, CS and JD); Drafting the first version of the manuscript (SW); Critical review of the manuscript (AHD, DC, MM, JN, LB, BJH, LB-S, JS, TE, MG, MW, SR, SO, BT, SN, RL, AS-B, CS and JD).

Abbreviations

AE	Adverse event
ANOVA	Analysis of variance
CRF	Case report form
DHRA	Department of Health Risk Assessment
DOAC	Direct-acting oral anticoagulant
DVT	Deep vein thrombosis
ECTU	Edinburgh Clinical Trials Unit
EHIT	Endothermal heat-induced thrombosis
GCP	Good clinical practice
HAT	Hospital-acquired thrombosis

For peer review only - http://bmjopen.bmj.com/site/about/guidelines.xhtml

HEAP	Health economics analysis plan
HRA	Health Regulatory Authority
HTA	Health Technology Assessment
ICER	Incremental cost-effectiveness ratio
iDMC	Independent Data Monitoring Committee
LMWH	Low-molecular weight heparin
MHRA	Medicines and Healthcare Regulatory Agency
NHS	National Health Service
NICE	National Institute for Care and Excellence
NIHR	National Institute for Health Research
NNT	Number needed to treat
PE	Pulmonary embolism
PI	Principal Investigator
PPI	Patient and public involvement
PTS	Post-thrombotic syndrome
QALY	Quality adjusted life years
RAT	Risk assessment tool
RCT	Randomised controlled trial
REDCap	Research Electronic Data Capture
REC	Research Ethics Committee
SAE	Serious adverse event
SAP	Statistical analysis plan
SMS	Short message service
TSC	Trial Steering Committee
UK	United Kingdom
VTE	Venous thromboembolism

For peer review only

For peer review only - http://bmjopen.bmj.com/site/about/guidelines.xhtml

Figure legends

Figure 1. THRIVE study flow chart

Abbreviations: DHRA, Department of Health Risk Assessment; DOAC, direct-acting oral anticoagulant; DVT, deep vein thrombosis; ICER, incremental cost-effectiveness ratio; LMWH, low-molecular weight heparin; VTE, venous thromboembolism.

REFERENCES

1. Salim S, Machin M, Patterson BO, Onida S, Davies AH. Global Epidemiology of Chronic Venous Disease: A Systematic Review With Pooled Prevalence Analysis. Annals of Surgery. 2021 Dec;274(6):971.

2. Carradice D, Mazari F a. K, Samuel N, Allgar V, Hatfield J, Chetter IC. Modelling the effect of venous disease on quality of life. Br J Surg. 2011 Aug;98(8):1089–98.

3. Kurz X, Lamping DL, Kahn SR, Baccaglini U, Zuccarelli F, Spreafico G, et al. Do varicose veins affect quality of life? Results of an international population-based study. J Vasc Surg. 2001 Oct;34(4):641–8.

4. Adam DJ, Naik J, Hartshorne T, Bello M, London NJM. The diagnosis and management of 689 chronic leg ulcers in a single-visit assessment clinic. Eur J Vasc Endovasc Surg. 2003 May;25(5):462–8.

5. Overview | Varicose veins: diagnosis and management | Guidance | NICE [Internet]. NICE; 2013 [cited 2023 Jun 2]. Available from: https://www.nice.org.uk/guidance/cg168

6. De Maeseneer MG, Kakkos SK, Aherne T, Baekgaard N, Black S, Blomgren L, et al. Editor's Choice - European Society for Vascular Surgery (ESVS) 2022 Clinical Practice Guidelines on the Management of Chronic Venous Disease of the Lower Limbs. Eur J Vasc Endovasc Surg. 2022 Feb;63(2):184–267.

7. Barwell JR, Davies CE, Deacon J, Harvey K, Minor J, Sassano A, et al. Comparison of surgery and compression with compression alone in chronic venous ulceration (ESCHAR study): randomised controlled trial. Lancet. 2004 Jun 5;363(9424):1854–9.

8. Gohel MS, Heatley F, Liu X, Bradbury A, Bulbulia R, Cullum N, et al. A Randomized Trial of Early Endovenous Ablation in Venous Ulceration. N Engl J Med. 2018 May 31;378(22):2105–14.

For peer review only

For peer review only - http://bmjopen.bmj.com/site/about/guidelines.xhtml

9. Brittenden J, Cotton SC, Elders A, Ramsay CR, Norrie J, Burr J, et al. A randomized trial comparing treatments for varicose veins. N Engl J Med. 2014 Sep 25;371(13):1218–27.

10. Rasmussen LH, Lawaetz M, Bjoern L, Vennits B, Blemings A, Eklof B. Randomized clinical trial comparing endovenous laser ablation, radiofrequency ablation, foam sclerotherapy and surgical stripping for great saphenous varicose veins. Br J Surg. 2011 Aug;98(8):1079–87.

11. Cai PL, Hitchman LH, Mohamed AH, Smith GE, Chetter I, Carradice D. Endovenous ablation for venous leg ulcers. Cochrane Database Syst Rev. 2023 Jul 27;7(7):CD009494.

12. Carradice D, Mekako AI, Mazari F a. K, Samuel N, Hatfield J, Chetter IC. Clinical and technical outcomes from a randomized clinical trial of endovenous laser ablation compared with conventional surgery for great saphenous varicose veins. Br J Surg. 2011 Aug;98(8):1117–23.

13. Carradice D, Mekako AI, Mazari F a. K, Samuel N, Hatfield J, Chetter IC. Randomized clinical trial of endovenous laser ablation compared with conventional surgery for great saphenous varicose veins. Br J Surg. 2011 Apr;98(4):501–10.

14. NHS Digital [Internet]. [cited 2023 Jul 19]. Hospital Admitted Patient Care Activity, 2021-22. Available from: https://digital.nhs.uk/data-and-information/publications/statistical/hospital-admitted-patient-care-activity/2021-22

15. Braekkan SK, Grosse SD, Okoroh EM, Tsai J, Cannegieter SC, Naess IA, et al. Venous thromboembolism and subsequent permanent work-related disability. J Thromb Haemost. 2016 Oct;14(10):1978–87.

16. NHS Digital [Internet]. 2022 [cited 2023 Sep 5]. Deaths from venous thromboembolism (VTE) related events within 90 days post discharge from hospital. Available from: https://digital.nhs.uk/data-and-information/publications/statistical/nhs-outcomes-framework/march-2022

17. Kahn SR, Shapiro S, Wells PS, Rodger MA, Kovacs MJ, Anderson DR, et al. Compression stockings to prevent post-thrombotic syndrome: a randomised placebo-controlled trial. Lancet. 2014 Mar 8;383(9920):880–8.

18. Eklof B, Perrin M, Delis KT, Rutherford RB, Gloviczki P, American Venous Forum, et al. Updated terminology of chronic venous disorders: the VEIN-TERM transatlantic interdisciplinary consensus document. J Vasc Surg. 2009 Feb;49(2):498–501.

19. Noble S, Lewis R, Whithers J, Lewis S, Bennett P. Long-term psychological consequences of symptomatic pulmonary embolism: a qualitative study. BMJ Open. 2014 Apr 1;4(4):e004561.

20. Barco S, Russo M, Vicaut E, Becattini C, Bertoletti L, Beyer-Westendorf J, et al. Incomplete echocardiographic recovery at 6 months predicts long-term sequelae after intermediate-risk pulmonary embolism. A post-hoc analysis of the Pulmonary Embolism Thrombolysis (PEITHO) trial. Clin Res Cardiol. 2019 Jul;108(7):772–8.

21. Bouras G, Burns EM, Howell AM, Bottle A, Athanasiou T, Darzi A. Risk of Post-Discharge Venous Thromboembolism and Associated Mortality in General Surgery: A Population-Based Cohort Study Using Linked Hospital and Primary Care Data in England. PLoS One. 2015;10(12):e0145759.

For peer review only - http://bmjopen.bmj.com/site/about/guidelines.xhtml

22. Whittley S, Machin M, Burgess L, Onida S, Carradice D, Davies A. Thromboprophylaxis strategies in patients undergoing endovenous thermal ablation: a UK survey. JVascSocGBIrel [Internet]. 2023 [cited 2023 Nov 16];3. Available from: https://jvsgbi.com/thromboprophylaxis-strategies-in-patients-undergoing-endovenous-thermal-ablation-a-uk-survey/

23. Boyle E, Reid J, O'Donnell M, Harkin D, Badger S. Thromboprophylaxis for varicose vein procedures - A national survey. Phlebology. 2019 Oct;34(9):598–603.

24. Turner BRH, Machin M, Jasionowska S, Salim S, Onida S, Shalhoub J, et al. Systematic Review and meta-analysis of the Additional Benefit of Pharmacological Thromboprophylaxis for Endovenous Varicose Vein Interventions. Ann Surg. 2022 Sep 15;

25. Recommendations | Venous thromboembolism in over 16s: reducing the risk of hospital-acquired deep vein thrombosis or pulmonary embolism | Guidance | NICE [Internet]. NICE; 2018 [cited 2023 Jun 7]. Available from: https://www.nice.org.uk/guidance/ng89/chapter/Recommendations

26. Dattani N, Shalhoub J, Nandhra S, Lane T, Abu-Own A, Elbasty A, et al. Reducing the risk of venous thromboembolism following superficial endovenous treatment: A UK and Republic of Ireland consensus study. Phlebology. 2020 Oct 1;35(9):706–14.

27. Schulman S, Kearon C, Subcommittee on Control of Anticoagulation of the Scientific and Standardization Committee of the International Society on Thrombosis and Haemostasis. Definition of major bleeding in clinical investigations of antihemostatic medicinal products in non-surgical patients. J Thromb Haemost. 2005 Apr;3(4):692–4.

28. Hwang IK, Shih WJ, De Cani JS. Group sequential designs using a family of type I error probability spending functions. Stat Med. 1990 Dec;9(12):1439–45.

29. Sweetland S, Green J, Liu B, Berrington de González A, Canonico M, Reeves G, et al. Duration and magnitude of the postoperative risk of venous thromboembolism in middle aged women: prospective cohort study. BMJ. 2009 Dec 3;339:b4583.

30. Curtis L, Burns A. Unit Costs of Health and Social Care 2017 [Internet]. Personal Social Services Research Unit, University of Kent; 2017 [cited 2023 Oct 23]. Available from: https://doi.org/10.22024/UniKent/01.02/65559

31. British National Formulary. Tinzaparin sodium, Medicinal forms [Internet]. 2022 [cited 2023 Oct 23]. Available from: https://bnf.nice.org.uk/drugs/tinzaparin-sodium/medicinal-forms/

32. Enden T, Resch S, White C, Wik HS, Kløw NE, Sandset PM. Cost-effectiveness of additional catheter-directed thrombolysis for deep vein thrombosis. J Thromb Haemost. 2013 Jun;11(6):1032–42.

33. NICE. Guide to the methods of technology appraisal (PMG9) [Internet]. NICE; 2023 [cited 2023 Nov 14]. Available from: https://www.nice.org.uk/process/pmg9/chapter/foreword

34. Husereau D, Drummond M, Augustovski F, de Bekker-Grob E, Briggs AH, Carswell C, et al. Consolidated Health Economic Evaluation Reporting Standards 2022 (CHEERS 2022) statement: updated reporting guidance for health economic evaluations. BMC Medicine. 2022 Jan 12;20(1):23.

For peer review only - http://bmjopen.bmj.com/site/about/guidelines.xhtml

For peer review only

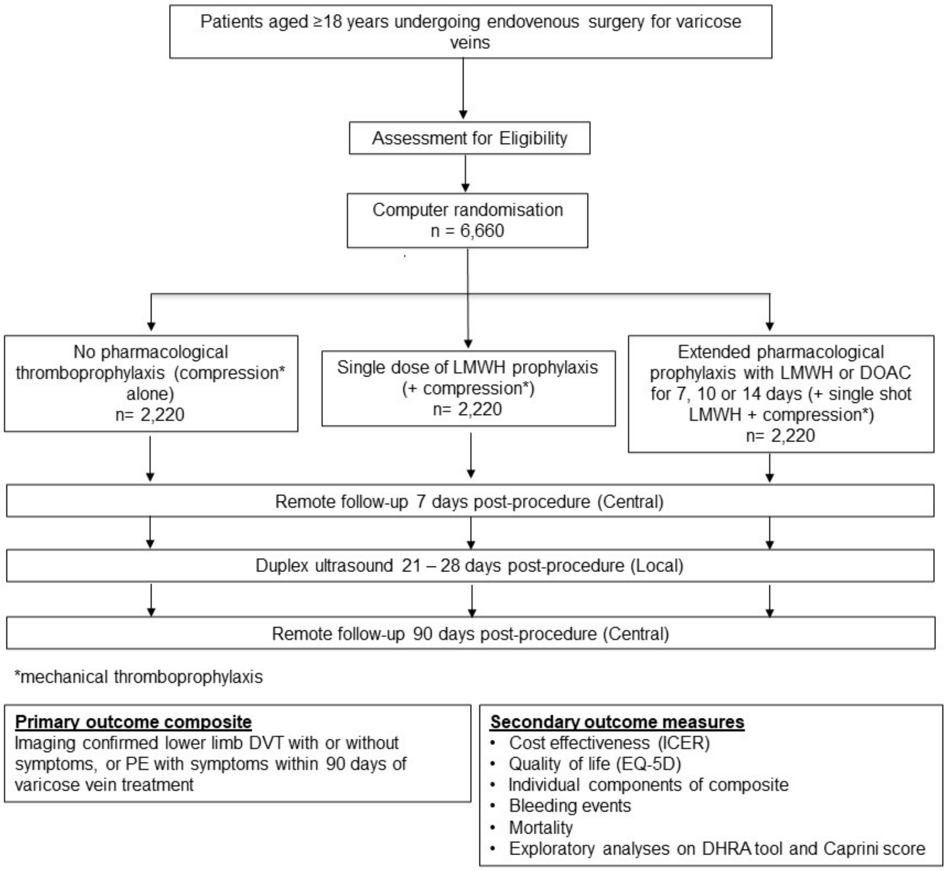

THRIVE study flow chart
Abbreviations: DHRA, Department of Health Risk Assessment; DOAC, direct-acting oral anticoagulant; DVT, deep vein thrombosis; ICER, incremental cost-effectiveness ratio; LMWH, low-molecular weight heparin; VTE, venous thromboembolism.

239x210mm (96 x 96 DPI)

Reporting checklist for protocol of a clinical trial.

Based on the SPIRIT guidelines.

Instructions to authors

Complete this checklist by entering the page numbers from your manuscript where readers will find each of the items listed below.

Your article may not currently address all the items on the checklist. Please modify your text to include the missing information. If you are certain that an item does not apply, please write "n/a" and provide a short explanation.

Upload your completed checklist as an extra file when you submit to a journal.

In your methods section, say that you used the SPIRITreporting guidelines, and cite them as:

Chan A-W, Tetzlaff JM, Gøtzsche PC, Altman DG, Mann H, Berlin J, Dickersin K, Hróbjartsson A, Schulz KF, Parulekar WR, Krleža-Jerić K, Laupacis A, Moher D. SPIRIT 2013 Explanation and Elaboration: Guidance for protocols of clinical trials. BMJ. 2013;346:e7586

For peer review only

		Reporting Item	Page Number
**Administrative information**			
Title	#1	Descriptive title identifying the study design, population, interventions, and, if applicable, trial acronym	1

Trial registration	#2a	Trial identifier and registry name. If not yet registered, name of intended registry	4
Trial registration: data set	#2b	All items from the World Health Organization Trial Registration Data Set	n/a
Protocol version	#3	Date and version identifier	1
Funding	#4	Sources and types of financial, material, and other support	20
Roles and responsibilities: contributorship	#5a	Names, affiliations, and roles of protocol contributors	21
Roles and responsibilities: sponsor contact information	#5b	Name and contact information for the trial sponsor	20
Roles and responsibilities: sponsor and funder	#5c	Role of study sponsor and funders, if any, in study design; collection, management, analysis, and interpretation of data; writing of the report; and the decision to submit the report for publication, including whether they will have ultimate authority over any of these activities	18
Roles and responsibilities: committees	#5d	Composition, roles, and responsibilities of the coordinating centre, steering committee, endpoint adjudication committee, data management team,	18

For peer review only

		and other individuals or groups overseeing the trial, if applicable (see Item 21a for data monitoring committee)	
**Introduction**			
Background and rationale	#6a	Description of research question and justification for undertaking the trial, including summary of relevant studies (published and unpublished) examining benefits and harms for each intervention	6
Background and rationale: choice of comparators	#6b	Explanation for choice of comparators	10
Objectives	#7	Specific objectives or hypotheses	8
Trial design	#8	Description of trial design including type of trial (eg, parallel group, crossover, factorial, single group), allocation ratio, and framework (eg, superiority, equivalence, non-inferiority, exploratory)	9
**Methods: Participants, interventions, and outcomes**			
Study setting	#9	Description of study settings (eg, community clinic, academic hospital) and list of countries where data	9

For peer review only

		will be collected. Reference to where list of study sites can be obtained	
Eligibility criteria	#10	Inclusion and exclusion criteria for participants. If applicable, eligibility criteria for study centres and individuals who will perform the interventions (eg, surgeons, psychotherapists)	9
Interventions: description	#11a	Interventions for each group with sufficient detail to allow replication, including how and when they will be administered	10
Interventions: modifications	#11b	Criteria for discontinuing or modifying allocated interventions for a given trial participant (eg, drug dose change in response to harms, participant request, or improving / worsening disease)	N/A – Standard of care; as per local practice
Interventions: adherance	#11c	Strategies to improve adherence to intervention protocols, and any procedures for monitoring adherence (eg, drug tablet return; laboratory tests)	n/a – pragmatic. Standard of care
Interventions: concomitant care	#11d	Relevant concomitant care and interventions that are permitted or prohibited during the trial	9
Outcomes	#12	Primary, secondary, and other outcomes, including the specific measurement variable (eg, systolic blood pressure), analysis metric (eg, change from baseline, final value, time to event), method of aggregation (eg, median, proportion), and time point	10

For peer review only

		for each outcome. Explanation of the clinical relevance of chosen efficacy and harm outcomes is strongly recommended	
Participant timeline	#13	Time schedule of enrolment, interventions (including any run-ins and washouts), assessments, and visits for participants. A schematic diagram is highly recommended (see Figure)	Figure 1
Sample size	#14	Estimated number of participants needed to achieve study objectives and how it was determined, including clinical and statistical assumptions supporting any sample size calculations	13
Recruitment	#15	Strategies for achieving adequate participant enrolment to reach target sample size	13
**Methods: Assignment of interventions (for controlled trials)**			
Allocation: sequence generation	#16a	Method of generating the allocation sequence (eg, computer-generated random numbers), and list of any factors for stratification. To reduce predictability of a random sequence, details of any planned restriction (eg, blocking) should be provided in a separate document that is unavailable to those who enrol participants or assign interventions	14

For peer review only

Allocation concealment mechanism	#16b	Mechanism of implementing the allocation sequence (eg, central telephone; sequentially numbered, opaque, sealed envelopes), describing any steps to conceal the sequence until interventions are assigned	14
Allocation: implementation	#16c	Who will generate the allocation sequence, who will enrol participants, and who will assign participants to interventions	13
Blinding (masking)	#17a	Who will be blinded after assignment to interventions (eg, trial participants, care providers, outcome assessors, data analysts), and how	14
Blinding (masking): emergency unblinding	#17b	If blinded, circumstances under which unblinding is permissible, and procedure for revealing a participant's allocated intervention during the trial	N/A
**Methods: Data collection, management, and analysis**			
Data collection plan	#18a	Plans for assessment and collection of outcome, baseline, and other trial data, including any related processes to promote data quality (eg, duplicate measurements, training of assessors) and a description of study instruments (eg, questionnaires, laboratory tests) along with their reliability and	14

For peer review only

		validity, if known. Reference to where data collection forms can be found, if not in the protocol	
Data collection plan: retention	#18b	Plans to promote participant retention and complete follow-up, including list of any outcome data to be collected for participants who discontinue or deviate from intervention protocols	12, 14
Data management	#19	Plans for data entry, coding, security, and storage, including any related processes to promote data quality (eg, double data entry; range checks for data values). Reference to where details of data management procedures can be found, if not in the protocol	14
Statistics: outcomes	#20a	Statistical methods for analysing primary and secondary outcomes. Reference to where other details of the statistical analysis plan can be found, if not in the protocol	15
Statistics: additional analyses	#20b	Methods for any additional analyses (eg, subgroup and adjusted analyses)	16
Statistics: analysis population and missing data	#20c	Definition of analysis population relating to protocol non-adherence (eg, as randomised analysis), and any statistical methods to handle missing data (eg, multiple imputation)	15

Methods: Monitoring

For peer review only

Data monitoring: formal committee	#21a	Composition of data monitoring committee (DMC); summary of its role and reporting structure; statement of whether it is independent from the sponsor and competing interests; and reference to where further details about its charter can be found, if not in the protocol. Alternatively, an explanation of why a DMC is not needed	18
Data monitoring: interim analysis	#21b	Description of any interim analyses and stopping guidelines, including who will have access to these interim results and make the final decision to terminate the trial	12
Harms	#22	Plans for collecting, assessing, reporting, and managing solicited and spontaneously reported adverse events and other unintended effects of trial interventions or trial conduct	18
Auditing	#23	Frequency and procedures for auditing trial conduct, if any, and whether the process will be independent from investigators and the sponsor	18
**Ethics and dissemination**			
Research ethics approval	#24	Plans for seeking research ethics committee / institutional review board (REC / IRB) approval	20

For peer review only

Protocol amendments	#25	Plans for communicating important protocol modifications (eg, changes to eligibility criteria, outcomes, analyses) to relevant parties (eg, investigators, REC / IRBs, trial participants, trial registries, journals, regulators)	20
Consent or assent	#26a	Who will obtain informed consent or assent from potential trial participants or authorised surrogates, and how (see Item 32)	13
Consent or assent: ancillary studies	#26b	Additional consent provisions for collection and use of participant data and biological specimens in ancillary studies, if applicable	N/A
Confidentiality	#27	How personal information about potential and enrolled participants will be collected, shared, and maintained in order to protect confidentiality before, during, and after the trial	14
Declaration of interests	#28	Financial and other competing interests for principal investigators for the overall trial and each study site	21
Data access	#29	Statement of who will have access to the final trial dataset, and disclosure of contractual agreements that limit such access for investigators	21
Ancillary and post trial care	#30	Provisions, if any, for ancillary and post-trial care, and for compensation to those who suffer harm from trial participation	N/A

For peer review only

Dissemination policy: trial results	#31a	Plans for investigators and sponsor to communicate trial results to participants, healthcare professionals, the public, and other relevant groups (eg, via publication, reporting in results databases, or other data sharing arrangements), including any publication restrictions	20
Dissemination policy: authorship	#31b	Authorship eligibility guidelines and any intended use of professional writers	20
Dissemination policy: reproducible research	#31c	Plans, if any, for granting public access to the full protocol, participant-level dataset, and statistical code	N/A

Appendices

Informed consent materials	#32	Model consent form and other related documentation given to participants and authorised surrogates	Yes
Biological specimens	#33	Plans for collection, laboratory evaluation, and storage of biological specimens for genetic or molecular analysis in the current trial and for future use in ancillary studies, if applicable	N/A

None The SPIRIT Explanation and Elaboration paper is distributed under the terms of the Creative Commons Attribution License CC-BY-NC. This checklist can be completed online using https://www.goodreports.org/, a tool made by the EQUATOR Network in collaboration with Penelope.ai
