# Supplementary material for: Evaluating pharmacological THRomboprophylaxis in Individuals undergoing superficial endoVEnous treatment across NHS and private clinics in the UK: a multi-centre, assessor-blind, randomised controlled trial—THRIVE trial
Source: BMJ Open. 2024 Feb 17;14(2):e083488. doi: 10.1136/bmjopen-2023-083488 (PMC10875503; doi:10.1136/bmjopen-2023-083488)
Supplement: Reviewer comments [file bmjopen-2023-083488.reviewer_comments.pdf]

PEER REVIEW HISTORY

BMJ Open publishes all reviews undertaken for accepted manuscripts. Reviewers are asked to complete a checklist review form (**http://bmjopen.bmj.com/site/about/resources/checklist.pdf**) and are provided with free text boxes to elaborate on their assessment. These free text comments are reproduced below.

ARTICLE DETAILS

**TITLE (PROVISIONAL)**	Evaluating pharmacological THRomboprophylaxis in Individuals undergoing superficial endoVEnous treatment across NHS and private clinics in the UK: a multi-centre, assessor blind, randomised controlled trial – THRIVE trial
**AUTHORS**	Machin, Matthew; Whittley, Sarah; Norrie, John; Burgess, Laura; Hunt, Beverley; Bolton, Layla; Shalhoub, Joseph; Everington, Tamara; Gohel, Manjit; Whiteley, Mark S; Rogers, Steven; Onida, Sarah; Turner, Benedict; Nandhra, Sandip; Lawton, Rebecca; Stephens-Boal, Annya; Singer, Carolyn; Dunbar, Joanne; Carradice, Daniel; Davies, AH

This article was not externally reviewed at BMJ Open. Protocols that have been independently assessed prior to submission to BMJ Open are usually fast-tracked to publication on the grounds that further substantial changes will not be possible. This independent assessment will usually be external, independent review for both a substantial grant award from a non-commercial or government funder and ethics approval.
